# The role of the RAS pathway in iAMP21-ALL

**DOI:** 10.1038/leu.2016.80

**Published:** 2016-05-10

**Authors:** S L Ryan, E Matheson, V Grossmann, P Sinclair, M Bashton, C Schwab, W Towers, M Partington, A Elliott, L Minto, S Richardson, T Rahman, B Keavney, R Skinner, N Bown, T Haferlach, P Vandenberghe, C Haferlach, M Santibanez-Koref, A V Moorman, A Kohlmann, J A E Irving, C J Harrison

**Affiliations:** 1 Northern Institute for Cancer Research, Newcastle University, Newcastle Upon Tyne, UK; 2 MLL Munich Leukemia Laboratory, Munich, Germany; 3 Bioinformatics Support Unit, Newcastle University, Newcastle Upon Tyne, UK; 4 Institute of Human Genetics, Newcastle University, Newcastle Upon Tyne, UK; 5 Institute of Cardiovascular Sciences, The University of Manchester, Manchester, UK; 6 Center for Human Genetics, University Hospital Leuven and KU Leuven, Leuven, Belgium; 7 AstraZeneca, Personalized Healthcare and Biomarkers, Innovative Medicines, Macclesfield, UK

## Abstract

Intrachromosomal amplification of chromosome 21 (iAMP21) identifies a high-risk subtype of acute lymphoblastic leukaemia (ALL), requiring intensive treatment to reduce their relapse risk. Improved understanding of the genomic landscape of iAMP21-ALL will ascertain whether these patients may benefit from targeted therapy. We performed whole-exome sequencing of eight iAMP21-ALL samples. The mutation rate was dramatically disparate between cases (average 24.9, range 5–51) and a large number of novel variants were identified, including frequent mutation of the RAS/MEK/ERK pathway. Targeted sequencing of a larger cohort revealed that 60% (25/42) of diagnostic iAMP21-ALL samples harboured 42 distinct RAS pathway mutations. High sequencing coverage demonstrated heterogeneity in the form of multiple RAS pathway mutations within the same sample and diverse variant allele frequencies (VAFs) (2–52%), similar to other subtypes of ALL. Constitutive RAS pathway activation was observed in iAMP21 samples that harboured mutations in the predominant clone (⩾35% VAF). Viable iAMP21 cells from primary xenografts showed reduced viability in response to the MEK1/2 inhibitor, selumetinib, *in vitro*. As clonal (⩾35% VAF) mutations were detected in 26% (11/42) of iAMP21-ALL, this evidence of response to RAS pathway inhibitors may offer the possibility to introduce targeted therapy to improve therapeutic efficacy in these high-risk patients.

## Introduction

Acute lymphoblastic leukaemia (ALL) is the most common childhood cancer. Chromosomal abnormalities define subgroups of ALL, often related to distinct clinical features and outcome.^1^ Recurrent copy number abnormalities (CNAs) and somatic mutations have been identified in genes within key cell-signalling pathways.^2, 3, 4, 5^ The involvement of different pathways is ALL subgroup specific, and their clinical and biological significance is related to the genomic background on which they occur.^3, 6, 7^ A range of inhibitors are being developed to target key signalling pathways, and experimental studies have shown response to these inhibitors in primary ALL cells.^4, 8, 9^ Thus, understanding the biological and clinical role of such abnormalities in individual ALL subgroups may have future therapeutic benefit.^10^

Patients with intrachromosomal amplification of chromosome 21 (iAMP21) comprise a distinct subgroup of childhood ALL at an incidence of 2%. They are older (median age 9 years) with a low white blood cell count (<50 × 10^9^/L) and have a high risk of relapse on standard therapy.^11^ Recently, we and others have shown that intensive treatment significantly improves outcome.^12, 13, 14, 15^ The abnormal chromosome 21 defining iAMP21 is highly heterogeneous with a complex array of rearrangements that affect copy number and localisation of many genes on chromosome 21.^16^ Although the mechanism behind the formation of this intriguing abnormality is understood, the target gene(s) and contributing leukaemogenic factor(s) remain unknown.

In this study, we explored the mutational landscape of iAMP21-ALL using next-generation sequencing technologies and discovered a high incidence of novel mutations, including frequent somatic mutation of genes within the RAS signalling pathway. Furthermore, we showed that MEK1/2 inhibition has a negative effect on viability of iAMP21-positive cells with phosphorylated ERK (pERK) expression. These findings suggest that RAS pathway deregulation is of biological relevance in iAMP21-ALL, which may offer alternative treatment approaches in these high-risk patients.

## Materials and methods

### Patient details

The study included diagnostic (*n*=44), matched remission (*n*=19) and/or relapse (*n*=7) bone marrow samples from ALL patients with iAMP21, and 48 and 66 diagnostic samples from patients with high hyperdiploidy (51–65 chromosomes) or no recurrent cytogenetic abnormality (B-other ALL), respectively (Supplementary Table 1). Studies were approved by the relevant institutional review boards, and written informed consent was obtained for each patient. DNA and RNA were extracted from bone marrow using the DNeasy and RNeasy Extraction kit (Qiagen, Manchester, UK), respectively. Whole-genome amplification of 20 ng DNA was performed using the Repli-G kit (Qiagen). A schema of the experimental approach is shown in Supplementary Figure 1.

### Cytogenetic analysis

Cytogenetic analysis and fluorescence *in situ* hybridisation were carried out, as previously reported.^17^ Patients were diagnosed with iAMP21-ALL, as described in Supplementary Table 1.

### SNP6.0 array analyses

SNP6.0 array analysis (Affymetrix, Santa Clara, CA, USA) was carried out on iAMP21-ALL cases with sufficient DNA: diagnostic (*n*=28) with matched relapse (*n*=2) and remission (*n*=17) (Supplementary Table 1). Arrays were prepared and analysed as described in Supplementary Table 2.

### Multiplex Ligation-dependent Probe Amplification

The SALSA MLPA P335-*IKZF1* kit (MRC Holland, Amsterdam, The Netherlands)was used to assess the copy number of 8 significant ALL-associated genes in 38 diagnostic and 6 relapse samples, as previously described.^18^ Matched SNP6.0 array data were available for 22 diagnostic and 1 relapse sample.

### Whole-exome sequencing

Whole-exome sequencing (WES) was performed on matched diagnostic and remission samples of eight iAMP21-ALL patients. Library preparation, sequencing, data analysis and validation of putative mutations were performed as described in Supplementary Text.

### Targeted sequencing approaches

Specific RAS pathway genes (*BRAF*, *NRAS*, *KRAS*, *FLT3*, *PTPN11* and *NF1*) were sequenced in a cohort of diagnostic (*n*=42), relapse (*n*=7) and remission (*n*=9) iAMP21-ALL, diagnostic B-other (*n*=66) and high hyperdiploid (*n*=48) samples, using amplicon sequencing (Supplementary Table 1). Primer sequences are detailed in Supplementary Table 3. Targeted sequencing was performed in two screens (A and B), as described in Supplementary Figure 1. Amplicon libraries were sequenced on the Roche 454 GS FLX or GS Junior sequencer at the Munich Leukemia Laboratory (Munich, Germany) or NewGene (Newcastle upon Tyne, UK) and on the Illumina MiSeq by Source Bioscience (Nottingham, UK). Data analysis was performed using SeqNext version 3.4.0 (JSI Medical Systems, Kippenheim, Germany), and mutations were identified by manual assessment using criteria described in Supplementary Figure 1.

### Reverse transcription PCR

The iScript cDNA Synthesis Kit (Bio-Rad, Hercules, CA, USA) was used to synthesise cDNA from 100 ng RNA. Primers were designed to exons 13 and 16 of *FLT3* (Supplementary Table 3). PCR was performed using peqGOLD Taq DNA polymerase (Peqlab, Erlangen, Germany).

### Western blotting

Protein was extracted from bone marrow samples and cell lines to assess levels of pERK (catalogue #4370, New England BioLabs, Ipswich, MA, USA) relative to ERK (sc-153, Santa Cruz Biotechnology, Dallas, TX, USA), as previously described.^5^ Two iAMP21-ALL samples (patients 1 and 7) and six positive/negative control samples (four patient samples and two cell lines, REH and CCRF-CEM) were used in western blotting analysis. Cell lines were obtained from European Collection of Cell Cultures or American Tissue Culture Collection and were authenticated and tested for mycoplasma contamination before use.

### Cytotoxic assessment of MEK1/2 inhibitors

Primary leukaemia cells from four iAMP21-ALL (patients 1, 14, 45, and 46b) were successfully transplanted into female NOD/LtSz-scid IL2Rγ null (NSG) mice, as previously described.^19^ Animal experiments were carried out in accordance with the UK Home Office Animals (Scientific Procedures) Act (ASPA) 1986 under project licence PPL60/4552. RAS gene mutations were screened in the diagnostic (patients 1 and 14) and/or primagraft (patients 1, 14, 45 and 46b) material, based on DNA availability. Viable leukaemia cells from the primagrafts were used to assess the cytotoxicity of the MEK1/2 inhibitor, selumetinib, as detailed previously.^8^

## Results

### WES and genomic analysis of iAMP21-ALL

WES identified 199 somatic mutations in 8 iAMP21-ALL samples that were predicted to be deleterious to protein function (Supplementary Table 4). All mutations selected for validation (*n*=106) were confirmed to be somatically acquired (Supplementary Text). The number of mutations was variable in each patient sample, ranging from 5 to 51 (average, 24.9), and on average the mutated allele was represented in 33% (range 4–100%) of reads; this value was designated the variant allele frequency (VAF). Most of the mutations were novel (73%, 146/199) and not previously reported in cancer (Catalogue of Somatic Mutations in Cancer, COSMIC). Substitutions, insertions and/or deletions were identified, which generated missense, in-frame, frameshift and splice site variations. Recurrent mutations were identified in four genes; *NRAS*, *CAPN5*, *FLT3LG* and *DOCK11*, in two samples each. Interestingly, nine mutations were detected in components of the RAS pathway in 75% (6/8) of iAMP21-ALL samples. Six mutations had been previously reported in cancer, *NRAS*, p.G12S (*n*=2), *FLT3LG* pQ114fs (*n*=2), *FLT3* p.N676K and *PTPN11* p.A72T; three mutations were novel, *RASGRP3* p.E445K, *NF1* p.P1667S and *CBL* p.E113Q. The average VAF of RAS pathway mutations was 31% (range 6–67%). Four samples harboured mutations that were representative of the major clone (⩾35% VAF).

The type of substitution (for example: transition, C>T) and sequence context in which it arises can define a ‘mutational signature', which can be created by an endogenous or environmental factor.^20, 21^ In the iAMP21-ALL exome, transitions were more common than transversions or indels, and 186 nucleotide substitutions were observed, predominantly composed of C:G>T:A substitutions that were enriched at CpG or TpC sites (Supplementary Figure 2).

### RAS pathway abnormalities are common in iAMP21-ALL

**Figure 1:**
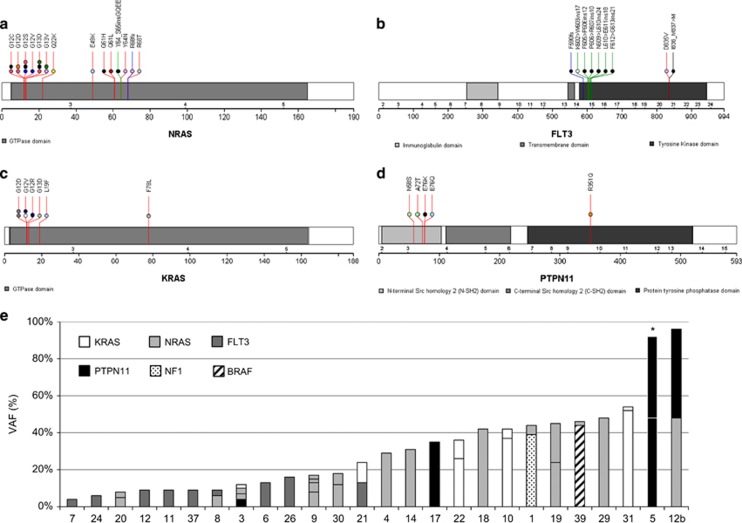
The nature and incidence of RAS pathway mutations in iAMP21-ALL. (**a–d**) Protein domain and alteration plots for RAS pathway genes that harbour mutations in 26 iAMP21-ALL samples: 44 mutations were identified in *NRAS* (**a**), *FLT3* (B), *KRAS* (**c**) and *PTPN11* (**d**). Each coloured line depicts the type of mutation (mismatch (red), deletion (black), insertion (green) and frameshift (blue)), and the amino-acid change is stated above the position/line. Most of the mutations had been previously reported in cancer (COSMIC), were frequently located in hotspot mutation regions within the gene and often observed as internal tandem duplications (ITD) in *FLT3*. The coloured circles represent the 26 individual samples; those cases with a single RAS pathway mutation are coloured black and each sample with multiple mutations is defined by a distinct colour. (**e**) The co-occurring nature of RAS pathway mutations is shown in 26 iAMP21-ALL patient samples. Each sample is labelled on the x axis and the y axis defines the VAF (%) of each mutation. The pattern/colour of each bar represents the mutated gene, as depicted by the key. *PTPN11 p.N58S, present in the matched diagnosis and remission sample. The protein domain and alteration plots were generated using The Protein Painter application in the Pediatric Cancer Genome Project (http://explore.pediatriccancergenomeproject.org).

Discovery of this high incidence of RAS pathway mutations by WES, prompted us to investigate the true incidence of RAS pathway mutations in iAMP21-ALL. From screening of 42 diagnostic and 5 relapse samples using targeted sequencing approaches (Supplementary Figure 1), distinct mutations (*n*=44) were identified in 26 samples overall (Figure 1, Supplementary Figure 3 and Supplementary Table 5), with a lower frequency at relapse (20%, 1/5) than diagnosis (60%, 25/42). Most mutations (89%, 39/44) had been previously reported in cancer (COSMIC) and all variants were consistently predicted to alter protein function. The mutational profile differed between diagnosis and relapse in seven matched samples (Supplementary Table 6). Testing of matched remission material (*n*=9) confirmed the somatic nature of the mutations in eight patients (Supplementary Table 1); however, *PTPN11* p.N58S was identified as a heterozygous variant in both diagnostic and remission samples of one patient (Figure 1e and Supplementary Figure 3). Germline mutations of *PTPN11* have been previously associated with Noonan and Leopard Syndromes, with an increased risk of developing cancer.^5, 22^

### RAS pathway abnormalities are genetically heterogeneous

The 44 mutations involved *NRAS* (45%, 20/44), *KRAS* (18%, 8/44), *FLT3* (20%, 9/44), *PTPN11* (11%, 5/44), *BRAF* (2%, 1/44) and *NF1* (2%, 1/44) (Figure 1 and Supplementary Figure 3). The RAS pathway mutations were shown to coexist within a gene or individual patient sample in patterns which ranged from 2–3 mutated genes to 2–4 mutations affecting the same gene in one sample. In eight cases, where one exon was affected by ⩾2 mutations, the variants were consistently observed in different read populations, indicating individual alleles or subclones, of which the latter would increase the overall mutational burden in these samples (Supplementary Figure 4). In one exception (patient 5), the germline variant, *PTPN11* p.N58S, and somatic mutation, *PTPN11* p.A72T, were identified within the same allele. The clonal origin of the mutations in different genes/exons could not be determined from the sequencing data in the remaining six cases, as they were located within different amplicons.

Consistent with this mutational heterogeneity, VAF differed between individual patient samples and RAS pathway genes (range 2–52%) (Figure 1 and Supplementary Figure 3). Overall, 26% (11/42) of diagnostic iAMP21-ALL cases harboured single or multiple RAS pathway mutations that, when combined, represented the major clone (total VAF ⩾35%); subclonal mutations were observed in 34% (14/42) of the cohort. *PTPN11* was frequently mutated at a higher frequency (average VAF, 35%) than *NRAS*, *KRAS*, or *FLT3*, which were often mutated in ⩽18% of reads. The single *NF1* and *BRAF* mutations were detected at levels representative of the dominant clone (⩾35%). Interestingly, *FLT3* was most frequently exclusively mutated (78%, 7/9 cases), whereas *NRAS*, *KRAS* and *PTPN11* rarely represented isolated mutations (⩽31% cases). The cohort was too small to determine the significance of these observations.

### Detection of RAS pathway mutations in other ALL subgroups

**Figure 2:**
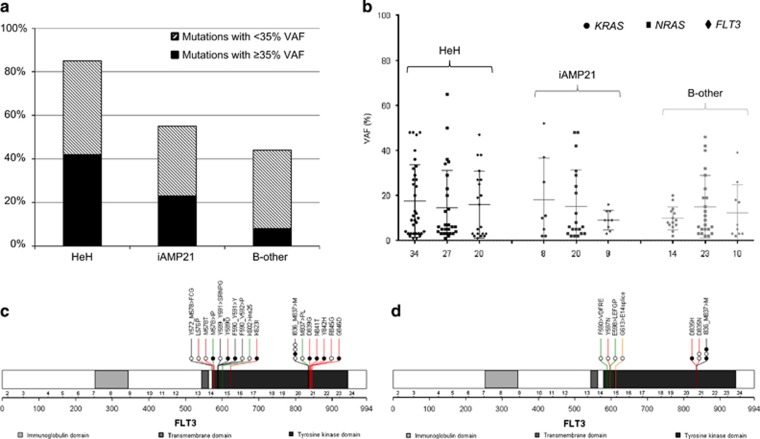
Nature of RAS pathway mutations in other ALL subgroups. *KRAS*, *NRAS* and *FLT3* were sequenced in patient samples with high hyperdiploid (*n*=48) or B-other ALL (*n*=66). Only exonic regions that were mutated in the iAMP21-ALL cohort were assessed. (**a**) The incidence of mutations in high hyperdiploid, iAMP21 and B-other ALL ranged from 44 to 85% the proportion of cases with significant mutational burden (⩾35% VAF (black bar)) or subclonal mutation (<35% VAF (patterned bar)) is shown. (**b**) VAF of *KRAS*, *NRAS* and *FLT3* mutations in high hyperdiploid, iAMP21 and B-other ALL is demonstrated. Subclonal mutations were identified for each gene, independent of ALL subgroup. (**c**, **d**) Protein domain plots for *FLT3* mutations in 17 high hyperdiploid (**c**) (20 mutations) and 5 B-other (**d**) (10 mutations) patient samples. Each coloured line depicts the type of mutation (mismatch (red), deletion (black), insertion (green) and splice site mutation (gold)), and the amino-acid change is stated above the position/line. The coloured circles represent individual samples; those cases with a single RAS pathway mutation are coloured black and samples with multiple mutations are defined by a white circle.

To investigate the distinct genetic profile of RAS pathway mutations in iAMP21-ALL, the same screening approach was performed on samples from high hyperdiploid (*n*=48) and B-other ALL patients (*n*=66). RAS pathway gene mutations were frequent in these subgroups, occurring in 85% (41/48) and 44% (29/66) of cases, respectively (Figure 2 and Supplementary Figure 5). These subgroups were selected for comparative analysis with iAMP21-ALL because they are known to have a high incidence of RAS pathway mutations, acquire extra copies of chromosome 21 (high hyperdiploidy) or have a similar gene expression profile (B-other).^3, 9, 23, 24^

Comparable patterns of genetic heterogeneity and subclonal *KRAS*, *NRAS* and *FLT3* mutations were identified in both subgroups (Figure 2 and Supplementary Figure 5). Significant mutational burden (VAF ⩾35%) was observed in 42% (20/48) and 8% (5/66) of high hyperdiploid and B-other ALL, respectively, compared with 26% (11/42) of diagnostic iAMP21-ALL. *FLT3* mutations were frequently found at a lower VAF in all subgroups, but tended to arise independently of other RAS pathway gene mutations only in iAMP21-ALL. *FLT3* mutation types ranged from substitutions to indels and varied according to the ALL subgroup in which they occurred; while substitutions and indels were common in high hyperdiploid and B-other ALL, *FLT3*-ITD was significantly more prevalent in iAMP21-ALL (*χ*^2^ test, ITD versus non-ITD in iAMP21 and non-iAMP21-ALL, *P*=0.0001).

### CNAs affecting RAS pathway genes

**Figure 3:**
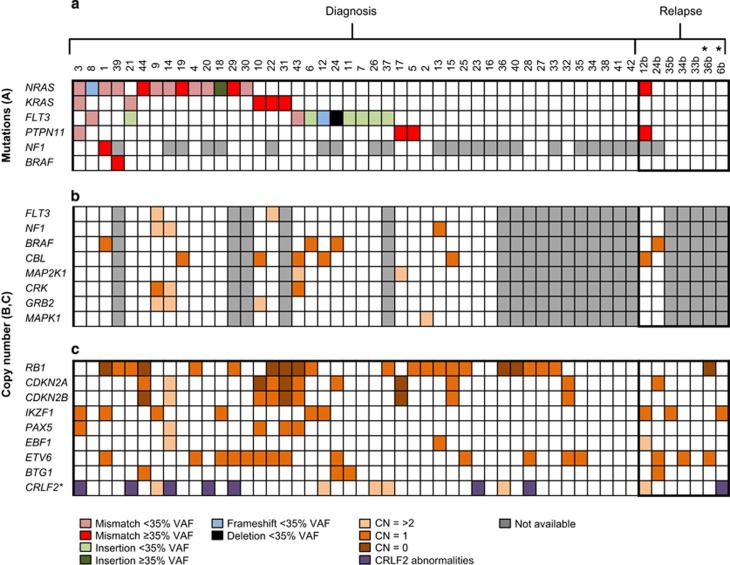
The association between RAS pathway mutations and CNAs in iAMP21-ALL. iAMP21-ALL patient samples (*n*=49, 42 diagnostic and 7 relapse) were screened for (**a**) RAS pathway mutations, (**b**) RAS pathway CNAs by SNP6.0 array analysis of 30 iAMP21-ALL cases (28 diagnostic and 2 relapse samples) and (**c**) CNA that affects genes recurrently aberrant in ALL. *Two relapse cases were used to validate the diagnostic-specific nature of RAS gene mutations, but they were not included in the mutation screening cohort. Grey denotes that the genomic assay was not performed. The type and VAF (%) of the mutation and the copy number (CN) status of the gene are represented by the respective colour. *CRLF2* abnormalities are shown in purple. Only genes that harboured mutations or CNA are displayed. CNA was frequently observed in genes (*BRAF*, *NF1*, *CBL*) located on chromosomes recurrently abnormal in iAMP21-ALL but they were not observed alongside mutations in cases analysed by targeted or whole-exome sequencing.^14^ There was a trend towards co-occurrence of *IKZF1* deletions and RAS mutations (Fisher's exact test, *P*=0.065).

To determine the relationship between CNA and RAS pathway mutations in iAMP21-ALL, SNP6.0 array and MLPA (Multiplex Ligation-dependent Probe Amplification) data were available for 30 (28 diagnostic and two relapse) and 44 (38 diagnostic and six relapse) samples, respectively, for which the mutational status of the RAS pathway was known. There was 100% concordance (42/42 abnormalities) between the MLPA and SNP6.0 array data in samples (*n*=22) assessed using both methods. *RB1* and *ETV6* deletions were frequent in 41% (20/49) and 33% (16/49) of cases, respectively, as previously shown.^18, 25^ CNA affecting components of the RAS pathway was identified in 53% (16/30) of cases but were not observed alongside gene mutations (Figure 3 and Supplementary Table 2). The CNA often involved large (>1 Mb) regions (95%, 20/21) of chromosomes recurrently abnormal in iAMP21-ALL, for example loss/deletion of chromosomes 7/7q (*BRAF* locus) and 11q (*CBL* locus).^14^ One focal CNA (75 kb) targeted only the *FLT3* locus (patient 9), although the other allele was not mutated. Regions of copy number neutral loss of heterozygosity (CNN-LOH) were not observed for RAS pathway genes among patients in this cohort. Although limited by small sample size, a trend was observed for an association between RAS pathway mutations and *IKZF1* deletions (Fisher's exact test, *P*=0.065).

### The biological significance and utility of RAS pathway mutations as therapeutic targets

**Figure 4:**
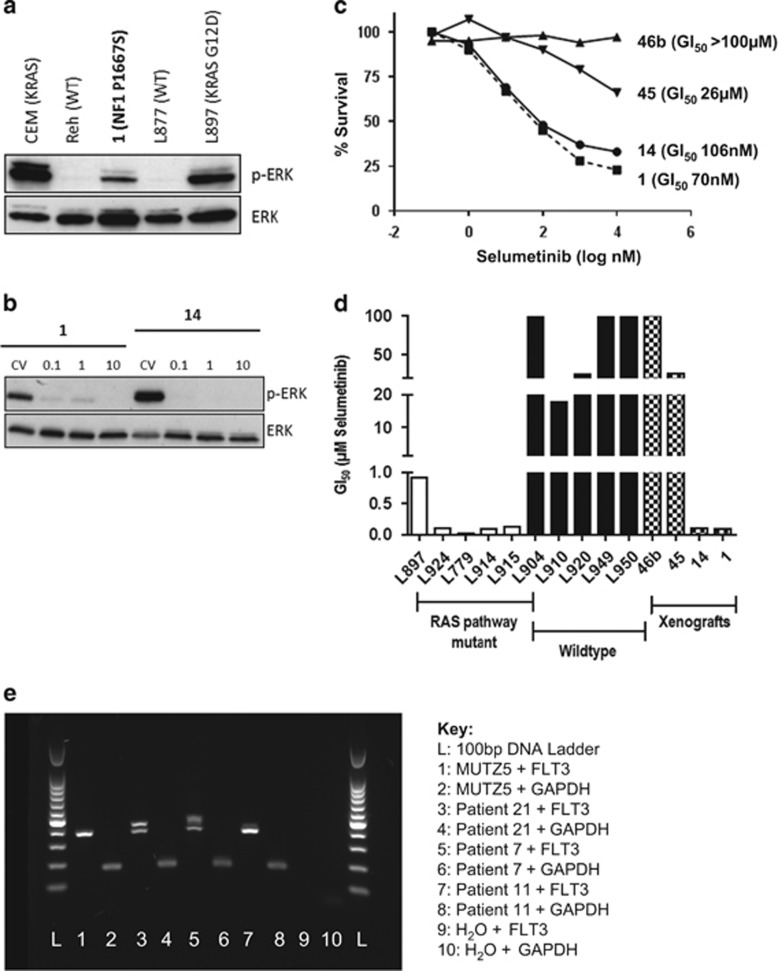
The biological significance of RAS pathway mutations in iAMP21-ALL. (**a**) Western blot analysis was used to investigate the biological effect of RAS pathway mutations in patient 1 (bold text), relative to positive (*KRAS* mutated) and negative (wild type (WT)) control samples from our previous studies.^5, 8^ pERK was observed in the positive control samples and patient 1, which harboured *NF1* p.P1667S and *NRAS* p.Q22K. Absent pERK was observed in the negative control samples. (**b**, **c**) Cytotoxicity assays were performed using the MEK1/2 inhibitor, selumetinib, on viable cells from the xenografts of patients 1, 14 and the xenograft material of two iAMP21-ALL patients that did not harbour RAS pathway mutations (patients 45 and 46b) (Supplementary Figure 6). (**b**) Reduced pERK levels confirmed the inhibitory effect of selumetinib on RAS pathway signalling exclusively in the mutated samples. pERK expression was not detected in the RAS normal xenograft samples (data not shown). (**c**) Sensitivity to selumetinib was observed in the RAS mutant samples (patients 1 and 14) only. (**d**) Bar chart of GI50 values after dosing with selumetinib for the xenografts of patients 1, 14, 45 and 46b, compared with RAS mutant and non-mutated ALL samples from our previous study.^8^ A similar level of sensitivity to selumetinib was observed in patients 1 and 14, compared with other RAS mutant ALL samples. Similarly, the response observed in patients 45 and 46b was equivalent to non-mutated ALL samples. (**e**) Transcript expression of *FLT3*-ITD and normal *FLT3* allele in patients 7, 11 and 21, as measured by RT-PCR. *FLT3*-ITD was expressed in all mutant cases with VAF of 13% (patient 21), 4% (patient 7) and 9% (patient 11). MUTZ5 was used as a control for normal *FLT3* expression. A 100-bp DNA ladder (Promega, Madison, WI, USA) was used to determine the PCR product size.

To elucidate the biological significance of RAS pathway mutations in iAMP21-ALL, protein analysis was performed to assess pERK levels in samples with sufficient material. Constitutive RAS pathway activation was confirmed in patient 1 (*NF1* p.P1667S and *NRAS* p.Q22K), although the pERK level was lower than the positive control samples (CEM and L897), which both harboured *KRAS* p.G12D mutations (Figure 4). Patient 7 (*FLT3* p.P606_R607ins10, VAF=4%) showed no detectable pERK, demonstrating that this *FLT3* mutation did not activate the RAS/RAF/ERK pathway, or at least not at detectable levels (Supplementary Figure 6). RT-PCR (reverse transcription PCR), performed on patient 7 and two additional iAMP21-ALL samples (patients 11 and 21), confirmed that the normal and mutant *FLT3* alleles were transcriptionally expressed in cases harbouring *FLT3*-ITD at 4–13% VAF (Figure 4e).

Therapeutic inhibitors of the RAS pathway are currently in clinical trial and have previously been shown to induce apoptosis in ALL cells with aberrant RAS pathway activation.^5, 8, 26^ To assess the therapeutic benefit of RAS/RAF/ERK pathway inhibitors in iAMP21-ALL, viable cells from xenografts of diagnostic leukaemia cells from RAS mutant patients 1 and 14 were tested. The same mutations identified in the diagnostic sample were present in the 1°, 2° and 3° xenograft material (Supplementary Figure 6). The 3° xenograft material from two other iAMP21-ALL patients negative for RAS pathway mutations was used as controls. Sensitivity to the MEK1/2 inhibitor, selumetinib (AZD6244), was observed *in vitro* in association with inhibition of RAS pathway signalling, evidenced by reduced pERK levels, in patients 1 and 14 only (Figure 4).

## Discussion

This is the first study of the mutational landscape of iAMP21-ALL. We have shown that the total number of mutations is variable (5–51 somatic mutations per case); higher than the average mutation rate in other ALL subtypes (iAMP21, 24.9; ETV6-RUNX1, 14; high hyperdiploidy, 7.5; hypodiploidy, 10.9; *MLL*-rearranged, infant 1.3, children 6.5).^4, 27, 28, 29^ The mutational signature can indicate the endogenous or environmental factor that is involved in the development of a specific disease.^20, 21^ The signature of iAMP21-ALL appeared to be similar to other ALL series, exhibiting a high rate of C>T substitutions at CpG and TpC sites.^21, 28, 30^ This signature is associated with DNA methylation; the most widespread mutational process in genome evolution and cancer. Novel recurrent mutations were identified in genes not previously associated with childhood ALL; *CAPN5*, *FLT3LG* and *DOCK11*, suggesting that some mutations involved in iAMP21-ALL may be unique to this subgroup.

Our initial novel discovery of RAS pathway mutations in 6/8 iAMP21-ALL patients by WES led to the finding of a high incidence (60%) of these mutations in iAMP21-ALL at diagnosis. This high incidence may reflect the sensitivity of the targeted sequencing assay for the detection of subclonal mutations (Supplementary Figure 1). However, recent studies using similar targeted sequencing approaches have reported comparable incidences and patterns of RAS mutations in other ALL subtypes.^4, 9, 24, 27, 29^ In this study, subclonal and coexisting RAS mutations were observed, similar to these in iAMP21-ALL, in high hyperdiploid and B-other ALL. Subclonal mutations (<35% VAF) that coexisted were consistently identified in distinct read populations, representing individual alleles or subclones. Single-cell genomic studies have demonstrated that subclonal *KRAS* and *NRAS* mutations are often present in distinct clones in other cancer types, and frequent convergence from multiple RAS mutated subclones at diagnosis to a single clonal RAS mutation at relapse has been reported in ALL.^24, 31, 32^ Considering these data, coexisting subclones of RAS mutations detected in this study likely represent individual subclones, which collectively increase the total mutational burden within a single sample. The observation that most cases had detectable subclonal mutations demonstrates their potentially important role in ALL development. The coexistence of a range of low frequency variants may offer multiple routes for leukaemia development. RAS pathway mutations have been variously described as mutually exclusive or coexistent within other cancer subtypes,^33^ indicating that coexisting mutations may be required to achieve neoplasia in specific tissues or environments. Furthermore, multiple subclones with different RAS pathway mutations may provide a mechanism by which the leukaemic cells evade the effects of treatment. Our previous studies demonstrated that RAS pathway mutations can drive relapse and generate a more chemo-resistant phenotype, supported by the enrichment of *KRAS* mutant cells during induction chemotherapy and the observation of RAS gene mutations consistently in the major clone at relapse.^8^ In this study, *NRAS* p.G12D and *PTPN11* p.R351Q were detected in one iAMP21-ALL relapse sample as heterozygous variants (48% VAF). Although they were not detected in the matched diagnostic sample, it is possible that they were present in a low level subclone. The role of RAS mutant subclones in driving relapse is not well understood, as demonstrated by lack of correlation in the genomic profile of seven matched diagnostic and relapse samples in our study.

In iAMP21-ALL, somatic mutations frequently occurred in hotspot regions of the RAS pathway, generating amino-acid changes in key domains predicted to affect the function of the protein. Accompanying loss of the normal allele, leading to homozygosity of the mutant allele, has been reported in other subtypes of ALL, including hypodiploidy.^4^ Although a high incidence of CNA (53%) was observed in components of the RAS pathway in iAMP21-ALL, they did not coexist with mutations, implying that homozygosity or CNN-LOH of RAS pathway genes is not important in this subtype. Most CNA involved large (>1 Mb) chromosomal regions that were recurrently abnormal in iAMP21-ALL, which included multiple genes.^14^ However, mutation of neighbouring genes within the regions of CNA was not observed from WES, implicating that loss or gain of the functional gene, rather than homozygosity of the mutant allele, is important in iAMP21-ALL leukaemogenesis.

Most of the RAS pathway gene mutations reported here have been previously described in other ALL or cancer types (http://cancer.sanger.ac.uk/cancergenome/projects/cosmic/). However, this is the first report of a *RASGRP3* mutation within a haematological malignancy. Further screening for *RASGRP3* and *NF1* mutations was not carried out due to their rarity (1/8 and 1/20 cases, respectively). A recurrent mutation (p.Q114fs) was identified by WES in the ligand, *FLT3LG* (25%, 2/8). Although *FLT3LG* mutations are rare in haematological malignancies and have not been previously reported in ALL, mutation of its receptor, *FLT3*, occurs in 10–15% of ALL.^3^ Both *FLT3LG* mutant samples harboured mutations in other RAS pathway genes (patient 3, *KRAS, NRAS* (*n*=2), *PTPN11*; patient 9, *NRAS* (*n*=4)), suggesting that, similar to other components of the RAS pathway, *FLT3LG* mutations coexist with other RAS gene mutations in iAMP21-ALL. Similarly, samples with these mutations may be responsive to inhibitors of the RAS pathway. The incidence and biological effect of *FLT3LG* mutations therefore requires further investigation in iAMP21-ALL. Moreover, the diversity of RAS pathway gene mutations observed in this study raises the question of optimum methods for mutation screening within a clinical setting. A wide genomic capture method, along with confirmation of activation of the pathway through pERK expression, may be the best future screening approach.

Although RAS gene mutations frequently co-occur in ALL, we showed here that *FLT3* mutations often occurred independently of other mutations (78% cases) in iAMP21-ALL. Furthermore, we identified a significantly higher incidence of *FLT3*-ITD compared with high hyperdiploid and B-other ALL, in which substitutions and indels were common. The mutated *FLT3* receptor constitutively activates a number of downstream signalling pathways implicated in leukaemogenesis (RAS/RAF/ERK, PI3K/AKT/mTOR and JAK/STAT5) (Supplementary Figure 7).^34, 35^ Despite the transcript expression from the *FLT3* mutant and normal alleles, pERK expression was negative in a patient with *FLT3*-ITD at 4% VAF. This observation may be a reflection of the lack of sensitivity of the assay or suggest activation of an alternative pathway. Considering *FLT3*-ITD has been associated with JAK/STAT pathway activation and is frequent in Ph-like ALL, in which patients show a high incidence of tyrosine kinase- and cytokine-signalling abnormalities, JAK/STAT pathway activation and share a similar gene expression profile to iAMP21-ALL, the mutant FLT3 receptor may have a specific biological role in iAMP21-ALL development.^6, 9, 35^ Therapeutic inhibitors are currently being considered for the treatment of ALL patients with JAK/STAT pathway deregulation or *FLT3*-ITD acute myeloid leukaemia (AML), although the mutation must be present in the major clone or overexpressed (in relation to the wild-type allele) in order to target the majority of the leukaemic cells. Although *FLT3*-ITD had an average VAF of 9% in iAMP21-ALL, this was not reflected in the transcript expression ratio of wild-type to mutant allele in iAMP21-ALL patients. The leukaemogenic potency and biological function of subclonal *FLT3-*ITD alleles that are overexpressed requires further investigation in order to explore the role of this abnormality in iAMP21-ALL and assess the utility of such novel targeted therapies in the treatment of this disease.

The same RAS mutation was retained in both the diagnostic and xenograft cells of two patient samples with significant mutation burden (*NF1* p.P1667S, VAF=44%, patient 1 xenograft and *NRAS* p.Q61H, VAF=47%, patient 14 xenograft). The xenograft cells showed sensitivity to the MEK1/2 inhibitor, selumetinib, *in vitro.* A cell line model is not available for iAMP21-ALL and, due to the rarity of this disease, only limited iAMP21-ALL xenograft material was available. We have previously shown that *in vitro* sensitivity of RAS pathway mutated ALL cells to selumetinib is mirrored in NSG mice engrafted with primary ALL cells, with both showing a dramatic reduction in viable cell numbers.^8^ Similarly, the xenograft cells of two iAMP21-ALL patients who did not harbour RAS pathway mutations were not sensitive to selumetinib and showed a comparable response to non-mutated samples from our previous study.^8^ Thus, the overall conclusion is that samples with significant RAS pathway mutation burden respond well to selumetinib. Although the clinical benefit of MEK inhibitors alone or in combination with other chemotherapeutic agents/targeted therapies is not well understood, clinical trials are ongoing in specific forms of RAS/RAF/ERK mutant cancer, including childhood ALL, to explore the most effective form of treatment and reduce the potential for treatment resistance, a problem that emerged in early studies of *BRAF* mutant malignant melanoma.^26, 36^ In our study, it was also apparent that (1) VAF of the mutation, (2) potency of the mutated gene in activating the RAS/RAF/ERK pathway, (3) type of mutation or biological function of the gene and (4) additional genomic abnormalities may influence the therapeutic response (Supplementary Figure 7). Certain genes and protein-coding mutations vary in their level of activation of the RAS/RAF/ERK pathway,^37, 38, 39^ as demonstrated by the variable pERK expression between RAS pathway mutated samples in this study. Furthermore, the importance of understanding the genomic landscape in a given sample was demonstrated by the response of xenograft cells from hypodiploid ALL patients that harboured *NRAS* and *NF1* (VAF >50%) mutations to PI3K/mTOR, but not MEK, inhibitors, despite the detection of pERK and pS6 levels.^4^ Although further comprehensive genomic and biological investigations are required to fully characterise those patients who may benefit from RAS/RAF/ERK pathway inhibition, here we indicate that there is emerging potential in response to MEK1/2 inhibition in iAMP21-ALL. These patients are currently treated with highly toxic, intensive therapy.^12, 14^ Therefore, the addition of MEK inhibitors into the treatment regimen of iAMP21-ALL patients with RAS pathway mutations in the major clone may enable modification of chemotherapy intensity.

In conclusion, this is the first study to explore the mutational landscape of iAMP21-ALL. A high incidence of RAS pathway mutations was observed against which targeted RAS/RAF/ERK pathway inhibitors may offer an additional therapeutic strategy to the current highly toxic chemotherapeutic agents given to these high-risk patients.

## References

[bib1] Harrison CJ, Johansson B. Acute Lymphoblastic Leukemia. 3rd edn. Wiley-Blackwell: New Jersey, USA, 2009.

[bib2] Mullighan CG, Goorha S, Radtke I, Miller CB, Coustan-Smith E, Dalton JD et al. Genome-wide analysis of genetic alterations in acute lymphoblastic leukaemia. Nature 2007; 446: 758–764. 17344859 10.1038/nature05690

[bib3] Zhang J, Mullighan CG, Harvey RC, Wu G, Chen X, Edmonson M et al. Key pathways are frequently mutated in high-risk childhood acute lymphoblastic leukemia: a report from the Children's Oncology Group. Blood 2011; 118: 3080–3087. 21680795 10.1182/blood-2011-03-341412 PMC3175785

[bib4] Holmfeldt L, Wei L, Diaz-Flores E, Walsh M, Zhang J, Ding L et al. The genomic landscape of hypodiploid acute lymphoblastic leukemia. Nat Genet 2013; 45: 242–252. 23334668 10.1038/ng.2532 PMC3919793

[bib5] Case M, Matheson E, Minto L, Hassan R, Harrison CJ, Bown N et al. Mutation of genes affecting the RAS pathway is common in childhood acute lymphoblastic leukemia. Cancer Res 2008; 68: 6803–6809. 18701506 10.1158/0008-5472.CAN-08-0101

[bib6] Roberts Kathryn G, Morin Ryan D, Zhang J, Hirst M, Zhao Y, Su X et al. Genetic Alterations Activating Kinase and Cytokine Receptor Signaling in High-Risk Acute Lymphoblastic Leukemia. Cancer Cell 2012; 22: 153–166. 22897847 10.1016/j.ccr.2012.06.005 PMC3422513

[bib7] Clappier E, Auclerc MF, Rapion J, Bakkus M, Caye A, Khemiri et al. An intragenic ERG deletion is a marker of an oncogenic subtype of B-cell precursor acute lymphoblastic leukemia with a favorable outcome despite frequent IKZF1 deletions. Leukemia 2014; 28: 70–77. 24064621 10.1038/leu.2013.277

[bib8] Irving J, Matheson E, Minto L, Blair H, Case M, Halsey C et al. Ras pathway mutations are prevalent in relapsed childhood acute lymphoblastic leukemia and confer sensitivity to MEK inhibition. Blood 2014; 124: 3420–3430. 25253770 10.1182/blood-2014-04-531871 PMC4246039

[bib9] Roberts KG, Li Y, Payne-Turner D, Harvey RC, Yang YL, Pei D et al. Targetable kinase-activating lesions in Ph-like acute lymphoblastic leukemia. N Engl J Med 2014; 371: 1005–1015. 25207766 10.1056/NEJMoa1403088 PMC4191900

[bib10] Weston BW, Hayden MA, Roberts KG, Bowyer S, Hsu J, Fedoriw G et al. Tyrosine kinase inhibitor therapy induces remission in a patient with refractory EBF1-PDGFRB-positive acute lymphoblastic leukemia. J Clin Oncol 2013; 31: e413–e416. 23835704 10.1200/JCO.2012.47.6770

[bib11] Moorman AV, Ensor HM, Richards SM, Chilton L, Schwab C, Kinsey SE et al. Prognostic effect of chromosomal abnormalities in childhood B-cell precursor acute lymphoblastic leukaemia: results from the UK Medical Research Council ALL97/99 randomised trial. Lancet Oncol 2010; 11: 429–438. 20409752 10.1016/S1470-2045(10)70066-8

[bib12] Moorman AV, Robinson H, Schwab C, Richards SM, Hancock J, Mitchell CD et al. Risk-directed treatment intensification significantly reduces the risk of relapse among children and adolescents with acute lymphoblastic leukemia and intrachromosomal amplification of chromosome 21: a comparison of the MRC ALL97/99 and UKALL2003 trials. J Clin Oncol 2013; 31: 3389–3396. 23940220 10.1200/JCO.2013.48.9377

[bib13] Heerema NA, Carroll AJ, Devidas M, Loh ML, Borowitz MJ, Gastier-Foster JM et al. Intrachromosomal amplification of chromosome 21 is associated with inferior outcomes in children with acute lymphoblastic leukemia treated in contemporary standard-risk children's oncology group studies: a report from the children's oncology group. J Clin Oncol 2013; 31: 3397–3402. 23940221 10.1200/JCO.2013.49.1308 PMC3770866

[bib14] Harrison CJ, Moorman AV, Schwab C, Carroll AJ, Raetz EA, Devidas M et al. An international study of intrachromosomal amplification of chromosome 21 (iAMP21): cytogenetic characterization and outcome. Leukemia 2014; 28: 1015–1021. 24166298 10.1038/leu.2013.317 PMC4283797

[bib15] Attarbaschi A, Mann G, Panzer-Grumayer R, Rottgers S, Steiner M, Konig M et al. Minimal residual disease values discriminate between low and high relapse risk in children with B-cell precursor acute lymphoblastic leukemia and an intrachromosomal amplification of chromosome 21: the Austrian and German acute lymphoblastic leukemia Berlin-Frankfurt-Munster (ALL-BFM) trials. J Clin Oncol 2008; 26: 3046–3050. 18565891 10.1200/JCO.2008.16.1117

[bib16] Li Y, Schwab C, Ryan SL, Papaemmanuil E, Robinson HM, Jacobs P et al. Constitutional and somatic rearrangement of chromosome 21 in acute lymphoblastic leukaemia. Nature 2014; 508: 98–102. 24670643 10.1038/nature13115 PMC3976272

[bib17] Harrison CJ, Moorman AV, Barber KE, Broadfield ZJ, Cheung KL, Harris RL et al. Interphase molecular cytogenetic screening for chromosomal abnormalities of prognostic significance in childhood acute lymphoblastic leukaemia: a UK Cancer Cytogenetics Group Study. Br J Haematol 2005; 129: 520–530. 15877734 10.1111/j.1365-2141.2005.05497.x

[bib18] Schwab CJ, Chilton L, Morrison H, Jones L, Al-Shehhi H, Erhorn et al. Genes commonly deleted in childhood B-cell precursor acute lymphoblastic leukemia: association with cytogenetics and clinical features. Haematologica 2013; 98: 1081–1088. 23508010 10.3324/haematol.2013.085175 PMC3696612

[bib19] Rehe K, Wilson K, Bomken S, Williamson D, Irving J, den Boer ML et al. Acute B lymphoblastic leukaemia-propagating cells are present at high frequency in diverse lymphoblast populations. EMBO Mol Med 2013; 5: 38–51. 23229821 10.1002/emmm.201201703 PMC3569652

[bib20] Alexandrov LB, Stratton MR. Mutational signatures: the patterns of somatic mutations hidden in cancer genomes. Curr Opin Genet Dev 2014; 24: 52–60. 24657537 10.1016/j.gde.2013.11.014 PMC3990474

[bib21] Alexandrov LB, Nik-Zainal S, Wedge DC, Aparicio SA, Behjati S, Biankin AV et al. Signatures of mutational processes in human cancer. Nature 2013; 500: 415–421. 23945592 10.1038/nature12477 PMC3776390

[bib22] Jongmans MC, van der Burgt I, Hoogerbrugge PM, Noordam K, Yntema HG, Nillesen WM et al. Cancer risk in patients with Noonan syndrome carrying a PTPN11 mutation. Eur J Hum Genet 2011; 19: 870–874. 21407260 10.1038/ejhg.2011.37 PMC3172922

[bib23] Paulsson K, Horvat A, Strombeck B, Nilsson F, Heldrup J, Behrendtz M et al. Mutations of FLT3, NRAS, KRAS, and PTPN11 are frequent and possibly mutually exclusive in high hyperdiploid childhood acute lymphoblastic leukemia. Genes Chromosomes Cancer 2008; 47: 26–33. 17910045 10.1002/gcc.20502

[bib24] Malinowska-Ozdowy K, Frech C, Schonegger A, Eckert C, Cazzaniga G, Stanulla M et al. KRAS and CREBBP mutations: a relapse-linked malicious liaison in childhood high hyperdiploid acute lymphoblastic leukemia. Leukemia 2015; 29: 1656–1667. 25917266 10.1038/leu.2015.107 PMC4530204

[bib25] Rand V, Parker H, Russell LJ, Schwab C, Ensor H, Irving J et al. Genomic characterization implicates iAMP21 as a likely primary genetic event in childhood B-cell precursor acute lymphoblastic leukemia. Blood 2011; 117: 6848–6855. 21527530 10.1182/blood-2011-01-329961

[bib26] Zhao Y, Adjei AA. The clinical development of MEK inhibitors. Nat Rev Clin Oncol 2014; 11: 385–400. 24840079 10.1038/nrclinonc.2014.83

[bib27] Paulsson K, Lilljebjorn H, Biloglav A, Olsson L, Rissler M, Castor et al. The genomic landscape of high hyperdiploid childhood acute lymphoblastic leukemia. Nat Genet 2015; 47: 672–676. 25961940 10.1038/ng.3301

[bib28] Papaemmanuil E, Rapado I, Li Y, Potter NE, Wedge DC, Tubio J et al. RAG-mediated recombination is the predominant driver of oncogenic rearrangement in ETV6-RUNX1 acute lymphoblastic leukemia. Nat Genet 2014; 46: 116–125. 24413735 10.1038/ng.2874 PMC3960636

[bib29] Andersson AK, Ma J, Wang J, Chen X, Gedman AL, Dang J et al. The landscape of somatic mutations in infant MLL-rearranged acute lymphoblastic leukemias. Nat Genet 2015; 47: 330–337. 25730765 10.1038/ng.3230 PMC4553269

[bib30] Lindqvist CM, Nordlund J, Ekman D, Johansson A, Moghadam BT, Raine et al. The mutational landscape in pediatric acute lymphoblastic leukemia deciphered by whole genome sequencing. Hum Mutat 2015; 36: 118–128. 25355294 10.1002/humu.22719 PMC4309499

[bib31] Melchor L, Brioli A, Wardell CP, Murison A, Potter NE, Kaiser MF et al. Single-cell genetic analysis reveals the composition of initiating clones and phylogenetic patterns of branching and parallel evolution in myeloma. Leukemia 2014; 28: 1705–1715. 24480973 10.1038/leu.2014.13

[bib32] Ma X, Edmonson M, Yergeau D, Muzny DM, Hampton OA, Rusch M et al. Rise and fall of subclones from diagnosis to relapse in pediatric B-acute lymphoblastic leukaemia. Nat Commun 2015; 6: 6604. 25790293 10.1038/ncomms7604 PMC4377644

[bib33] Kandoth C, McLellan MD, Vandin F, Ye K, Niu B, Lu C et al. Mutational landscape and significance across 12 major cancer types. Nature 2013; 502: 333–339. 24132290 10.1038/nature12634 PMC3927368

[bib34] Weisberg E, Barrett R, Liu Q, Stone R, Gray N, Griffin JD. FLT3 inhibition and mechanisms of drug resistance in mutant FLT3-positive AML. Drug Resist Updat 2009; 12: 81–89. 19467916 10.1016/j.drup.2009.04.001 PMC4472331

[bib35] Choudhary C, Brandts C, Schwable J, Tickenbrock L, Sargin B, Ueker et al. Activation mechanisms of STAT5 by oncogenic Flt3-ITD. Blood 2007; 110: 370–374. 17356133 10.1182/blood-2006-05-024018

[bib36] Caunt CJ, Sale MJ, Smith PD, Cook SJ. MEK1 and MEK2 inhibitors and cancer therapy: the long and winding road. Nat Rev Cancer 2015; 15: 577–592. 26399658 10.1038/nrc4000

[bib37] Cirstea IC, Gremer L, Dvorsky R, Zhang SC, Piekorz RP, Zenker M et al. Diverging gain-of-function mechanisms of two novel KRAS mutations associated with Noonan and cardio-facio-cutaneous syndromes. Hum Mol Genet 2013; 22: 262–270. 23059812 10.1093/hmg/dds426

[bib38] Grundler R, Miething C, Thiede C, Peschel C, Duyster J. FLT3-ITD and tyrosine kinase domain mutants induce 2 distinct phenotypes in a murine bone marrow transplantation model. Blood 2005; 105: 4792–4799. 15718420 10.1182/blood-2004-11-4430

[bib39] Steelman LS, Chappell WH, Abrams SL, Kempf RC, Long J, Laidler P et al. Roles of the Raf/MEK/ERK and PI3K/PTEN/Akt/mTOR pathways in controlling growth and sensitivity to therapy-implications for cancer and aging. Aging 2011; 3: 192–222. 21422497 10.18632/aging.100296 PMC3091517

