# Supplementary material for: The role of the RAS pathway in iAMP21-ALL
Source: Leukemia. 2016 May 10;30(9):1824–31. doi: 10.1038/leu.2016.80 (PMC5017527; doi:10.1038/leu.2016.80)
Supplement: Supplementary Information [file leu201680x1.pdf]

Supplementary Text

Whole exome sequencing

Matched diagnostic and remission DNA was available for eight iAMP21 patient samples. Exomes were captured using 3-5ug of DNA per sample and the Sureselect Human All Exon 50Mb V2 (patient 1) or V4 Kit (patients 3, 5, 7, 9, 21, 43 and 44), as recommended (Agilent Technologies, Santa Clara, USA). Each library was prepared for sequencing using the Paired-End Library Preparation Kit (Illumina, San Diego, California, USA). The diagnostic and remission exomes of patient 1 were each sequenced on two lanes of one flowcell on the Illumina Genome Analyzer IIX (GAIIX) using 75bp paired-end chemistry (Newcastle University, Newcastle upon Tyne, UK). The libraries of the remaining samples were sequenced on the Illumina HiSeq 2000 using 100bp paired-end chemistry (AROS Applied Biotechnology AS, Aarhus, Denmark). Average coverage was 60-fold (range, 28-fold to 100-fold) per patient sample, calculated using DepthOfCoverage (Genome Analysis Toolkit (GATK), Broad Institute).

Paired end reads of the diagnostic and remission sample were aligned to UCSC hg19 with appropriate read groups set using BWA MEM algorithm of Burrows-Wheeler Aligner (version 0.7.10-r789).[1] The resultant SAM alignments were then converted to co-ordinate sorted and indexed BAM files and de-duplicated using Picard tools (version 1.137) (http://picard.sourceforge.net/). Standard GATK sample preparation was used to perform indel realignment and base quality score recalibration on the BAM files (GATK version 3.4).[2,3] Haplotype Caller (HC) (GATK) was used to call variants in the BAM file of each sample according to the current GATK 3.4 JointGenotyping gVCF workflow. Individual de-duplicated and indel realigned matched diagnostic/remission BAM files were then merged into one BAM file which underwent a second round of indel realignment, followed by base quality score recalibration (using GATK 3.4), before undergoing joint tumour/normal variant calling using MuTect to identify diagnostic variants.[4]

Diagnostic-specific variants were annotated using Variant Effect Predictor (VEP).[5] Variants within coding exons or splice site regions were selected for further analysis. The functional effect of individual mutations was assessed using PolyPhen2 and SIFT, for which the scores were derived by VEP, and/or Mutation Taster.[6-8] COSMIC (Catalogue of Somatic Mutations in Cancer (COSMIC), version 66) and DBSNP (version 137) were used to characterise the variants which was outputted from VEP.[9, 10] Supplementary Table 4 contains a list of somatic mutations that were predicted to damage the function of the protein by the majority of prediction tools, when available for analysis (indels and splice site variations could often not be assessed using PolyPhen2 or SIFT). DBSNP variants with <1% minor allelic frequency (MAF) were included.

Code availability

The pipeline and associated scripts are available online through GitHub (https://github.com/MattBashton/MB-GATK-SGE).

Validation of putative mutations

53% (106/199) of candidate somatic mutations were validated using targeted sequencing approaches or Sanger sequencing (Supplementary Table 4).

Sanger sequencing validations were performed using WGA DNA of the diagnostic and remission sample. PCR products were generated using specific primers (Supplementary Table 3) and standard PCR conditions (AmpliTaq Gold® DNA polymerase, Applied Biosystems, Paisley, UK). Samples with a visible band by gel electrophoresis were purified using the QIAquick PCR Purification kit (Qiagen, Manchester, UK) and sent for Sanger sequencing (DBS Genomics, Durham University, Durham, UK). Sequencing traces were interpreted using FinchTV (Geospiza, Inc, Seattle, USA) and alignment was performed using BLAT (UCSC) and BLAST (NCBI).[11-13]

Targeted sequencing was performed using Haloplex Target Enrichment technology (Agilent Technologies). The assay was designed to capture regions surrounding the putative mutation using SureDesign (Agilent Technologies). Libraries were prepared using WGA DNA of the diagnostic and remission material from the eight iAMP21 patient samples, as recommended (Agilent Technologies). 16 samples were pooled together at equimolar concentrations and sequenced on the Illumina HiSeq 2000 using 100bp paired-end chemistry (Edinburgh Genomics, University of Edinburgh, UK). Data analysis and variant annotation was performed using the protocol that was established to analyse the whole exome sequencing libraries (read alignment using BWA, mutation calling following the GATK Best Practices workflow (HC, MuTect) and VEP to annotate the variants).

100% (106/106) of the putative mutations were validated and demonstrated to be somatic (i.e. no mutant reads were detected in the remission sample). None of the putative mutations that were selected for validation were undetected by the assay.

References

1 Li H, Durbin R. Fast and accurate long-read alignment with Burrows-Wheeler transform. Bioinformatics. 2010 Mar 1;26(5):589-95.

2 DePristo MA, Banks E, Poplin R, Garimella KV, Maguire JR, Hartl C, et al. A framework for variation discovery and genotyping using next-generation DNA sequencing data. Nature genetics. 2011 May;43(5):491-8.

3 Van der Auwera GA, Carneiro MO, Hartl C, Poplin R, Del Angel G, Levy-Moonshine A, et al. From FastQ data to high confidence variant calls: the Genome Analysis Toolkit best practices pipeline. Current protocols in bioinformatics / editoral board, Andreas D Baxevanis [et al]. 2013 Oct 15;11(1110):11 0 1- 0 33.

4 Cibulskis K, Lawrence MS, Carter SL, Sivachenko A, Jaffe D, Sougnez C, et al. Sensitive detection of somatic point mutations in impure and heterogeneous cancer samples. Nature biotechnology. 2013 Mar;31(3):213-9.

5 McLaren W, Pritchard B, Rios D, Chen Y, Flicek P, Cunningham F. Deriving the consequences of genomic variants with the Ensembl API and SNP Effect Predictor. Bioinformatics. 2010 Aug 15;26(16):2069-70.

6 Kumar P, Henikoff S, Ng PC. Predicting the effects of coding non-synonymous variants on protein function using the SIFT algorithm. Nature protocols. 2009;4(7):1073-81.

7 Adzhubei IA, Schmidt S, Peshkin L, Ramensky VE, Gerasimova A, Bork P, et al. A method and server for predicting damaging missense mutations. Nature methods. 2010 Apr;7(4):248-9.

8 Schwarz JM, Cooper DN, Schuelke M, Seelow D. MutationTaster2: mutation prediction for the deep-sequencing age. Nature methods. 2014 Apr;11(4):361-2.

9 Forbes SA, Beare D, Gunasekaran P, Leung K, Bindal N, Boutselakis H, et al. COSMIC: exploring the world's knowledge of somatic mutations in human cancer. Nucleic acids research. 2015 Jan;43(Database issue):D805-11.
10 Sherry ST, Ward MH, Kholodov M, Baker J, Phan L, Smigielski EM, et al. dbSNP: the NCBI database of genetic variation. Nucleic acids research. 2001 Jan 1;29(1):308-11.
11 Camacho C, Coulouris G, Avagyan V, Ma N, Papadopoulos J, Bealer K, et al. BLAST+: architecture and applications. BMC bioinformatics. 2009;10:421.
12 Kent WJ. BLAT--the BLAST-like alignment tool. Genome research. 2002 Apr;12(4):656-64.
13 Altschul SF, Gish W, Miller W, Myers EW, Lipman DJ. Basic local alignment search tool. Journal of molecular biology. 1990 Oct 5;215(3):403-10.

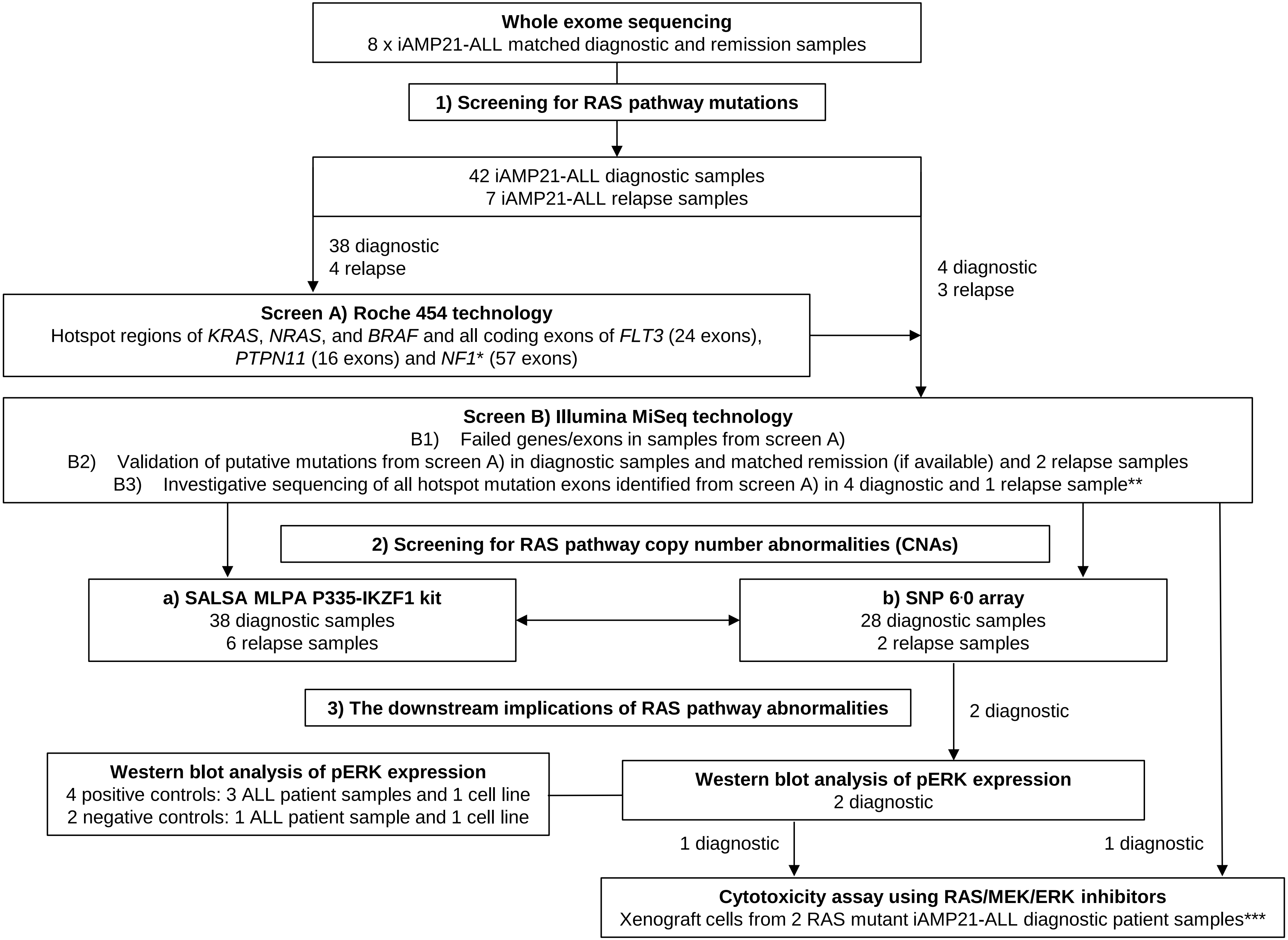
Whole exome sequencing
8 x iAMP21-ALL matched diagnostic and remission samples
1) Screening for RAS pathway mutations
42 iAMP21-ALL diagnostic samples
7 iAMP21-ALL relapse samples
38 diagnostic
4 relapse
4 diagnostic
3 relapse
Screen A) Roche 454 technology
Hotspot regions of KRAS, NRAS, and BRAF and all coding exons of FLT3 (24 exons), PTPN11 (16 exons) and NF1* (57 exons)
Screen B) Illumina MiSeq technology
B1) Failed genes/exons in samples from screen A)
B2) Validation of putative mutations from screen A) in diagnostic samples and matched remission (if available) and 2 relapse samples
B3) Investigative sequencing of all hotspot mutation exons identified from screen A) in 4 diagnostic and 1 relapse sample**
2) Screening for RAS pathway copy number abnormalities (CNAs)
a) SALSA MLPA P335-IKZF1 kit
38 diagnostic samples
6 relapse samples
b) SNP 6·0 array
28 diagnostic samples
2 relapse samples
3) The downstream implications of RAS pathway abnormalities
2 diagnostic
Western blot analysis of pERK expression
4 positive controls: 3 ALL patient samples and 1 cell line
2 negative controls: 1 ALL patient sample and 1 cell line
Western blot analysis of pERK expression
2 diagnostic
1 diagnostic
1 diagnostic
Cytotoxicity assay using RAS/MEK/ERK inhibitors
Xenograft cells from 2 RAS mutant iAMP21-ALL diagnostic patient samples***

Supplementary Figure 1: RAS pathway investigations in iAMP21-ALL. Whole exome sequencing was carried out on 8 matched diagnostic and remission samples, in which RAS pathway mutations were identified at an incidence of 75% (6/8).

(1) iAMP21-ALL samples (n=49, 42 diagnostic and 7 relapse) were subsequently screened for RAS pathway abnormalities. Two mutation screens were carried out: (A) screen A was performed using genomic DNA from 38 diagnostic and 4 relapse samples to investigate the mutational hotspot regions of *KRAS* (exons 2 and 3), *NRAS* (exons 2 and 3), and *BRAF* (exons 11 and 15) and all coding exons of *PTPN11*, *FLT3,* and *NF1* using Roche 454 sequencing technology. *Apart from patient 1, no other *NF1* mutations were identified in an additional 20 samples, thus no further cases were screened. Individual exons were amplified using a 4-primer PCR method; custom-made primers and Access Array Barcode Library primers (Fluidigm Technologies, San Francisco, California, USA) were used to generate individual PCR products using the Access Array System (Fluidigm Technologies) or ABI 2700 Thermocycler (Applied Biosystems, Foster City, California, USA). Amplicons were assessed on the Agilent Bioanalyser (DNA 1000 Assay, Agilent Technologies, Santa Clara, USA) and PCR products were pooled at equimolar concentrations. Data analysis was performed using version 3·4·0 SeqNext (JSI Medical Systems, Kippenheim, Germany) and mutations were identified by manual assessment. The average read coverage achieved for screen A was 418 reads (349-577 per gene). Putative mutations were selected for validation (screen B) if: i) present at a similar variant allele frequency (VAF) (% of mutated reads) and in ≥10 reads in both sequencing directions (forward and reverse) or ii) previously reported in COSMIC (http://cancer.sanger.ac.uk/cancergenome/projects/cosmic/) and iii) not in a region of homopolymer sequence. (B) Screen B was performed using whole genome amplified (WGA) DNA and employed Illumina MiSeq sequencing technology to: (B1) repeat any exons or genes that failed to sequence in screen A, (B2) validate the putative mutations identified in patient samples from screen A and (B3) identify mutations in the hotspot regions of *NRAS* (exons 2 and 3), *KRAS* (exons 2 and 3), *BRAF* (exons 11 and 15), *FLT3* (exons 5, 14, 15 and 20), and *PTPN11* (exons 3, 9, and 13) in an additional 4 diagnostic and 1 relapse iAMP21-ALL patient sample. Matched remission (n=9) and/or relapse (n=3) material was sequenced if sufficient material was available to confirm the somatic or diagnostic-specific nature of the mutations identified in Screen A. The average read coverage achieved for screen B was 35,280 (30,250-48,550 per gene). Mutations were confirmed/validated in screen B if present in ≥500 reads and at a similar VAF (%) to screen A. WGA DNA was representative of the original patient material, the VAF (%) and pattern of RAS mutations was similar for both sample derivatives. ** Genomic DNA of B-other (n=66) and high hyperdiploid (n=48) patient samples were screened for mutations in the hotspot regions of *NRAS* (exons 2 and 3), *KRAS* (exons 2 and 3) and *FLT3* (exons 4, 14, 15 and 20) using the same targeted sequencing approach. These genes/exons were selected due to their high mutation rate in iAMP21-ALL, to enable a comparison of mutational profiles between the three cytogenetic subgroups. Mutations were identified in the B-other and hyperdiploid patient samples if present in ≥50 reads and at a similar VAF (%) in both sequencing directions (forward and reverse).

(2) Copy number profiling was performed to assess the association between copy number abnormalities (CNAs) and mutations in the RAS pathway genes using (a) SALSA MLPA P335-IKZF1 kit on 38 diagnostic and 6 relapse iAMP21-ALL samples and (b) SNP6·0 analysis in 28 diagnostic and 2 relapse cases.

(3) The downstream functional effect of RAS pathway mutations was assessed in two patient samples and 4 positive controls (patient samples and cell lines) by western blot analysis of pERK expression. The cytotoxic effect of selumetinib (MEK1/2 pathway inhibitor) was tested using viable cells from the xenografts of primary cells from two iAMP21-ALL samples with RAS pathway mutations and positive pERK expression. *** Two iAMP21-ALL samples (patients 45 and 46b), in which the xenograft material was screened for RAS mutations, were used as controls in the cytotoxicity assay (Supplementary Figure 6). The results of these studies are reported in the manuscript.

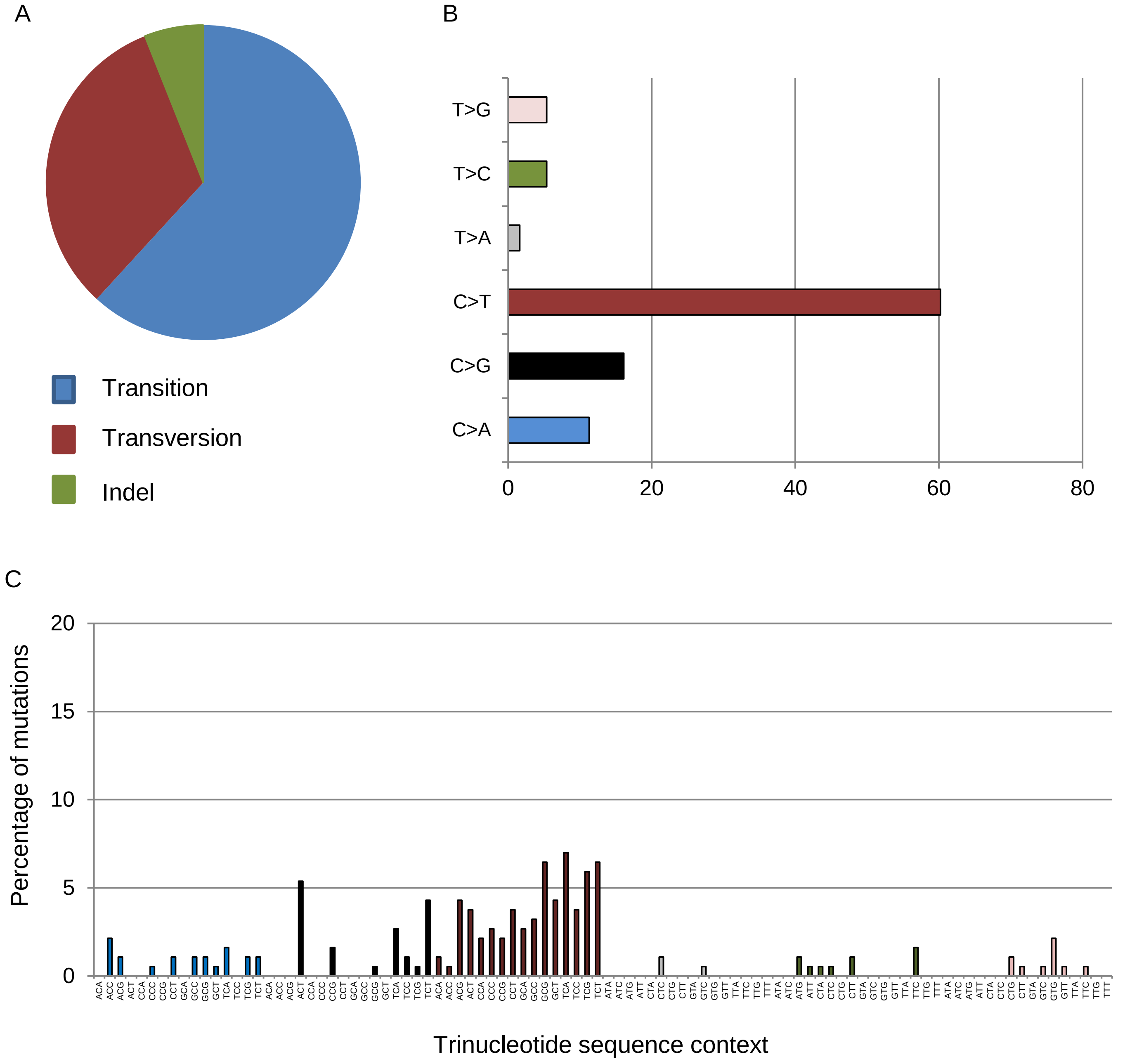

Supplementary Figure 2: The mutational landscape of iAMP21-ALL. The exome of 8 iAMP21-ALL patient samples harboured 186 nucleotide substitutions and 13 insertions or deletions (indels). (A) Transitions were more common than transversions (0.62:0.32) or indels (0.62: 0.06). (B) Mutations were predominantly composed of C:G>T:A substitutions (60%) with C:G>G:C and C:G>A:T mutations representing a smaller proportion (27%) of substitutions. (C) Most C>T substitutions tended to arise at CpG or TpC sites, a mutational signature that has previously been described in ALL and is associated with DNA methylation, an established process in genome evolution and cancer.[1,2]

1 Alexandrov LB, Nik-Zainal S, Wedge DC, Aparicio SA, Behjati S, Biankin AV, et al. Signatures of mutational processes in human cancer. Nature. 2013 Aug 22;500(7463):415-21.
2 Papaemmanuil E, Rapado I, Li Y, Potter NE, Wedge DC, Tubio J, et al. RAG-mediated recombination is the predominant driver of oncogenic rearrangement in ETV6-RUNX1 acute lymphoblastic leukemia. Nature genetics. 2014 Feb;46(2):116-25.

A

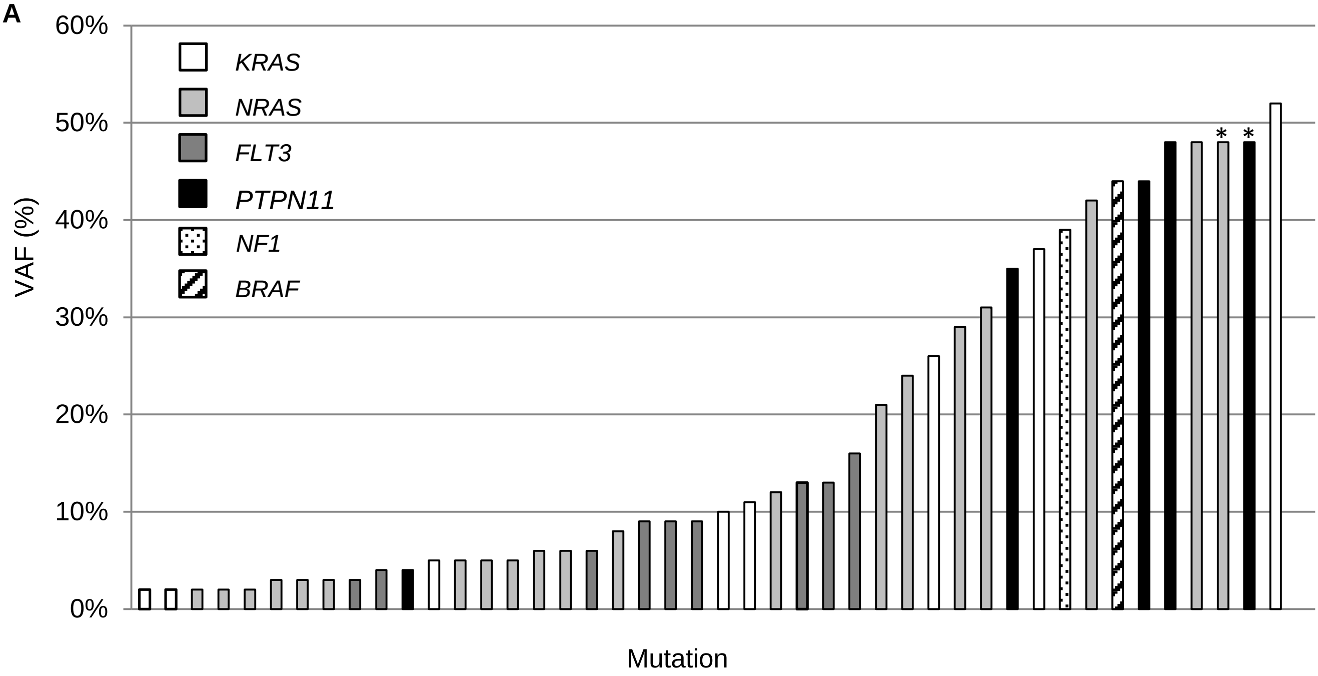

B

i) Gene	ii) # of mutations	iii) Average VAF (%)	iv) Co-occurring mutation rate (% of gene mutations that co-occurred with other RAS pathway mutations)
KRAS	8	15%	100.0%
NRAS	20	18%	69%
FLT3	9	9%	22%
PTPN11	5	35%	75%
NF1	1	39%	100%
BRAF	1	44%	100%

Supplementary Figure 3: The highly variable nature of RAS pathway mutations in iAMP21-ALL. 44 mutations were identified in components of the RAS pathway (*KRAS, NRAS, FLT3, PTPN11, NF1*, and *BRAF*) in 26 patient samples (25 diagnostic and 1 relapse (marked by *). Each RAS pathway gene is colour/pattern-coded as depicted in the respective figure. (A) The variant allele frequency (VAF (% of mutated reads / total reads at the base position)) of each RAS pathway mutation (n=44) identified in the iAMP21-ALL samples can be highly variable, ranging from 2-52%. Each bar represents a single mutation (x-axis) and the corresponding VAF is represented on the y-axis. (B) A tabular summary of the (ii) number of mutations reported for each gene, (iii) average VAF (%) of the mutations per gene and iv) proportion of cases that harboured ≥2 mutations (co-existent mutations) per mutated gene. *PTPN11*, *NF1* and *BRAF* were often mutated at a higher VAF (%) than other RAS pathway genes and *FLT3* mutations were present as single mutations more often than the other RAS pathway genes, however, the significance of these findings could not be assessed due to the small cohort size.

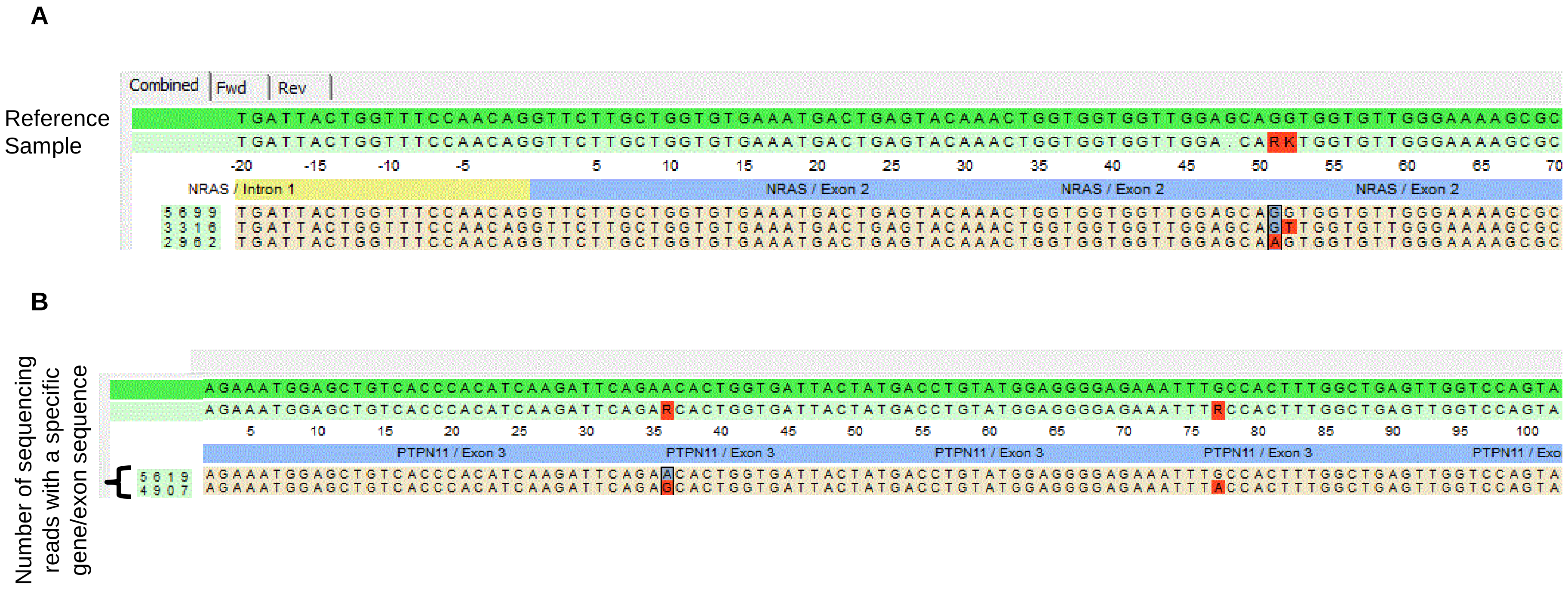

Supplementary Figure 4: RAS pathway mutations were often present in distinct read populations. Images were taken from SeqNext software (JSI Medical Systems) to demonstrate the subclonal nature of RAS pathway mutations in iAMP21-ALL patient samples 5 (A) and 19 (B). Reference genomic sequence and patient sample genomic sequence are shown. Mutations are highlighted in red text and the number of sequencing reads with a specific gene/exon sequence are depicted. (A) *NRAS* p.G12S and p.G12V mutations were detected in two individual read populations in patient 19; 2962 reads harboured the G->A substitution at base position 34 and 3316 reads had a G->T nucleotide change at base position 35, resulting in the p.G12S and p.G12V mutations, respectively. These two mutations were therefore present in different read populations and represented individual alleles or subclones, the latter of which would increase the mutational burden of RAS pathway mutations to 45% in this patient sample. Approximately half of the sequencing reads (n=5699) contained the normal sequence for *NRAS* exon 2. Similar findings were reported for 89% (8/9) of iAMP21-ALL samples, in which multiple mutations were identified within the same exon/amplicon. (B) *PTPN11* p.N58S (A->G at base position 173) and p.A72T (G->A at base position 214) were identified in the same read population (4907 reads) in patient 5, demonstrating that both mutations were present within the same allele.

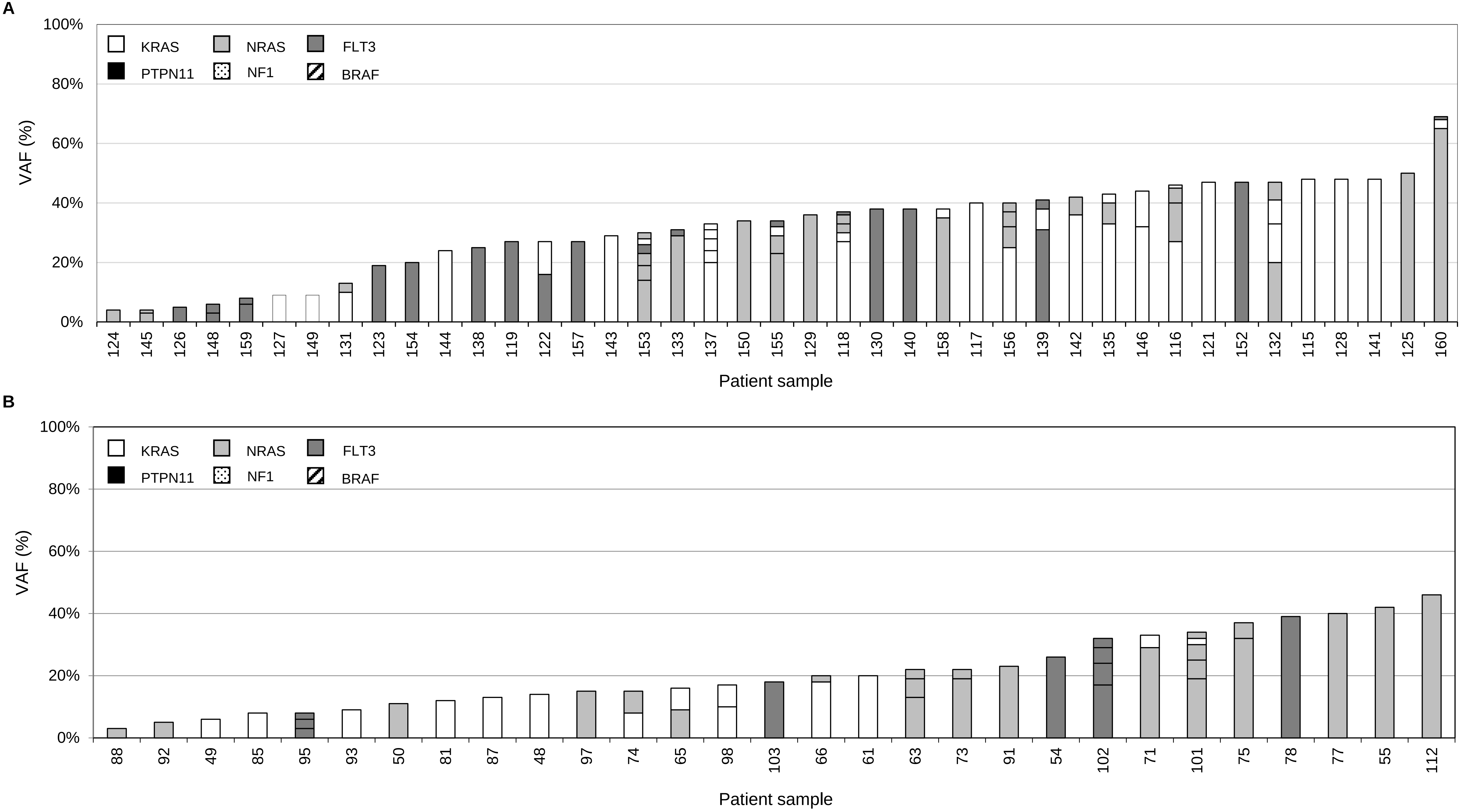

A
100%
80%
60%
40%
20%
0%
VAF (%)
KRAS
NRAS
FLT3
PTPN11
NF1
BRAF
124 145 126 148 159 127 149 131 123 154 144 138 119 122 157 143 153 133 137 150 155 129 118 130 140 158 117 156 139 142 135 146 116 121 152 132 115 128 141 125 160
Patient sample
B
100%
80%
60%
40%
20%
0%
VAF (%)
KRAS
NRAS
FLT3
PTPN11
NF1
BRAF
88 92 49 85 95 93 50 81 87 48 97 74 65 98 103 66 61 63 73 91 54 102 71 101 75 78 77 55 112
Patient sample

C

	Hyperdiploidy				iAMP21				B-other			
Gene	# of mutations	Incidence (%)	Average VAF (%)	Co-occurring mutation rate	# of mutations	Incidence (%)*	Average VAF (%)	Co-occurring mutation rate	# of mutations	Incidence (%)	Average VAF (%)	Co-occurring mutation rate
KRAS	34	71%	18%	74%	8	19%	15%	100.0%	14	21%	10%	50%
NRAS	27	56%	15%	85%	20	48%	18%	69%	23	35%	15%	65%
FLT3	20	42%	16%	55%	9	21%	9%	22%	6	9%	18%	50%

Supplementary Figure 5: The co-occurring nature of RAS pathway mutations in high hyperdiploid and B-other ALL. (A, B) *KRAS*, *NRAS* and *FLT3* mutations were investigated in patient samples with high hyperdiploid ALL (n=48) (A) or B-other ALL (n=66) (B). Each sample is labelled on the x-axis and the y-axis defines the variant allele frequency (VAF (%)) of each mutation. The pattern/colour of each bar represents the mutated gene, as depicted by the key. Subclonal mutations of *KRAS*, *NRAS* and *FLT3* were identified in hyperdiploid and B-other ALL. (C) The mutational profile of RAS pathway genes in ALL subgroups. The average VAF (%) and co-existence nature of *KRAS*, *NRAS* and *FLT3* mutations in high hyperdiploid (n=81), iAMP21- (n=37) and B-other ALL (n=43) that represent each gene are shown. Co-occurring mutation rate represents the % of gene mutations that co-occurred with another RAS pathway mutation. The incidence of gene mutations in the diagnostic sample of patients with high hyperdiploid (n=48) iAMP21- (n=42 (*not including relapse samples) and B-other (n=66) ALL; the co-existence of other RAS gene mutations in the same sample is not represented in this column. *FLT3* mutations tend to arise independently of other RAS pathway gene mutations in iAMP21-ALL (Fisher's exact test, isolated or co-existent *FLT3* mutations in iAMP21 and non-iAMP21-ALL, p=0.14).

A

Mutation	Sample ID	Diagnostic VAF(%)	Primary xenograft VAF (%)	Secondary xenograft VAF (%)	Tertiary xenograft VAF (%)
NF1 p.P1667S	1	39%	45%	48%	44%
NRAS p.Q22K	1	5%	6%	2%	7%
NRAS p.Q61H	14	31%	NA	47%	47%

B

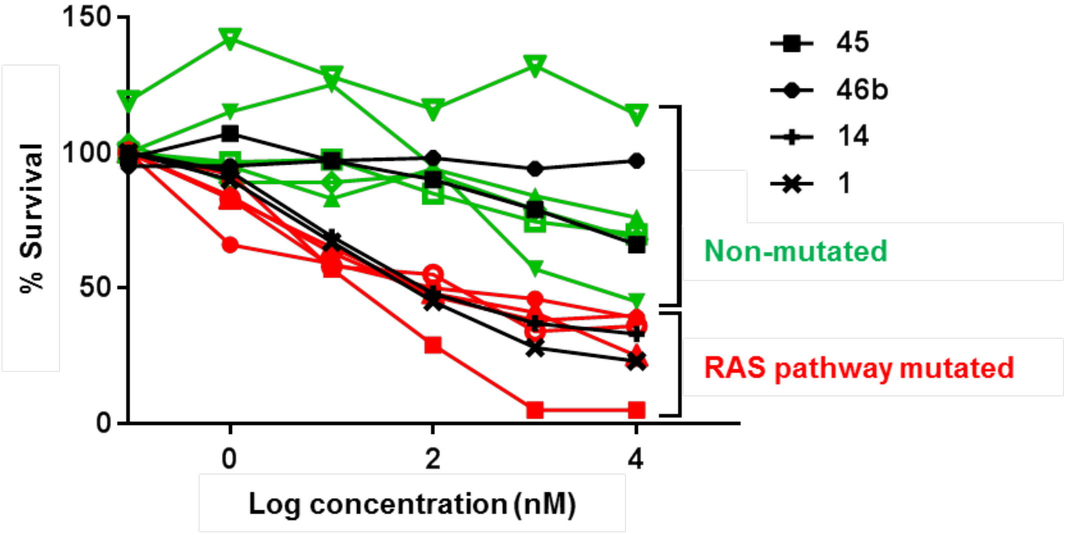

C

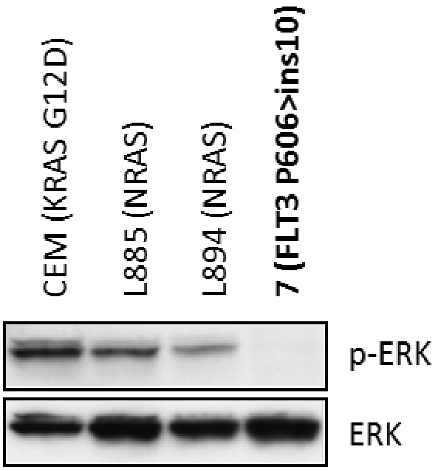

Supplementary Figure 6: RAS pathway mutations are maintained in 1^{O}, 2^{O}, and 3° xenograft models. (A) Summary of the sequencing data from the NGS targeted sequencing method. Mutations were assessed as discussed in Methods, Targeted Sequencing Approaches, of the main article. Three RAS pathway mutations were identified in the diagnostic material of patient 1 (n=2) and 14 (n=1). Variant allele frequency (VAF) represents the proportion of combined forward and reverse reads that harbour the mutation at the respective base position. Mutations were confirmed in the 1^{O}, 2^{O}, and 3° xenograft material and at similar variant allele frequencies to the diagnostic sample. *NRAS* (exons 2 and 3), *KRAS* (exons 2 and 3), *FLT3* (exons 14, 15 and 20) and *PTPN11* (exons 3, 9 and 13) mutations were not identified in the xenograft material of patients 45 or 46b. These RAS pathway genes/exons were specifically selected for sequencing as they were recurrently mutated in iAMP21 ALL patient samples. Xenograft cells from these patients were used as a control to assess the sensitivity of unmutated cells to selumetinib. (B) Cytotoxicity assays were performed using the MEK1/2 inhibitor, selumetinib, on viable cells from the xenografts of patients 1, 14 , 45 and 46b. Reduced cell viability was observed in response to selumetinib *in vitro* in patient 1 and 14 only, comparable to levels observed in other RAS mutant ALL samples (red) from our previous study.[1] Similarly, the response seen in patients 45 and 46b was equivalent to other non-mutated ALL samples (green). (C) pERK expression was assessed in patient 7 (bold text), relative to three RAS mutant samples. pERK was observed in the positive control samples and absent in patient 7, which harboured *FLT3*-ITD (p.P606_R607ins10, VAF=4%), demonstrating that this *FLT3* mutation did not activate the RAS/RAF/ERK pathway, or at least not to detectable levels .

References

1. Irving J, Matheson E, Minto L, Blair H, Case M, Halsey C, et al. Ras pathway mutations are prevalent in relapsed childhood acute lymphoblastic leukemia and confer sensitivity to MEK inhibition. Blood. 2014 Nov 27;124(23):3420-30.

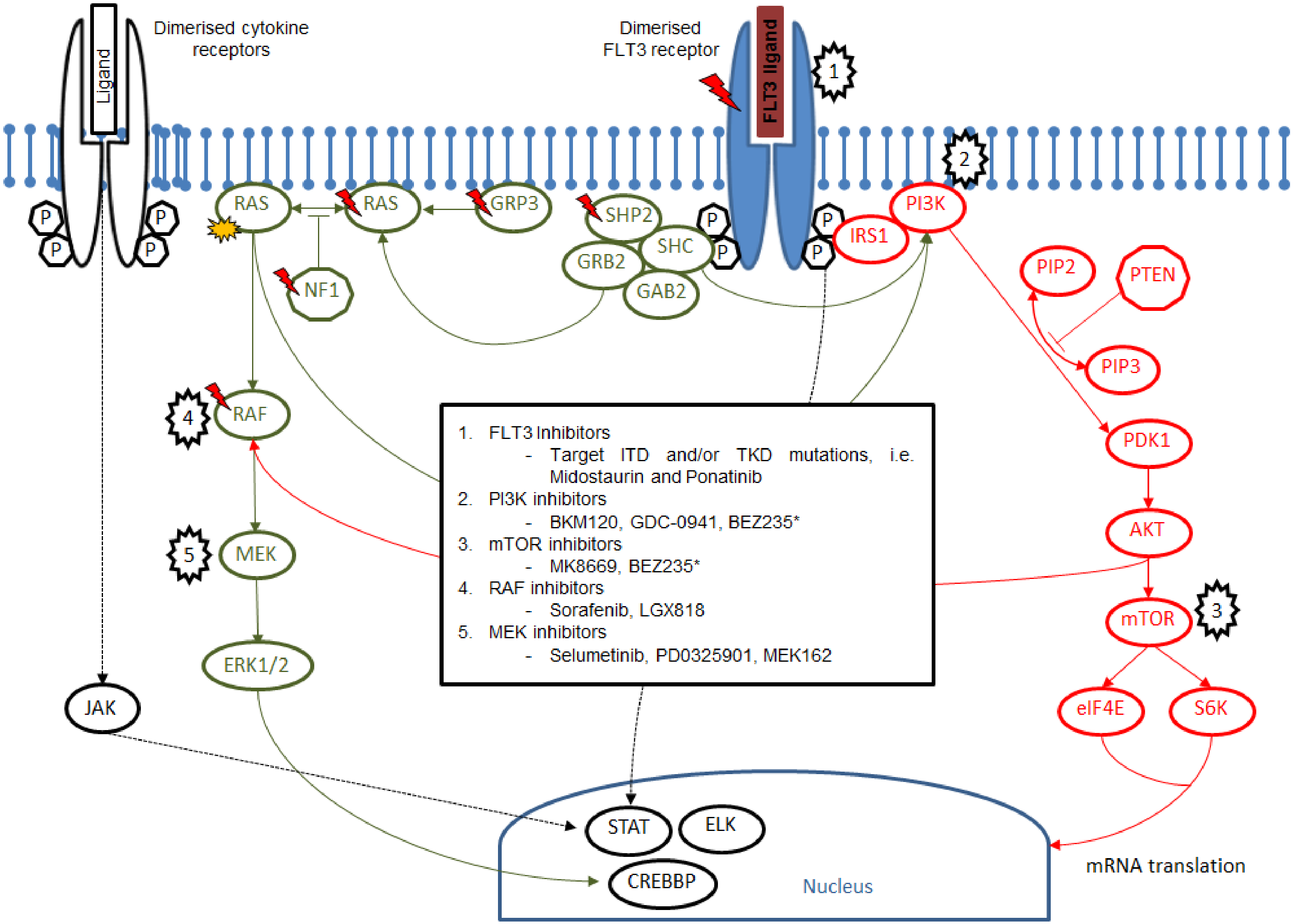
Dimerised cytokine receptors
Ligand
Dimerised FLT3 receptor
FLT3 ligand
1
2
3
4
5
P
RAS
RAS
GRP3
SHP2
SHC
GRB2
GAB2
NF1
IRS1
PI3K
PIP2
PTEN
PIP3
PDK1
AKT
mTOR
eIF4E
S6K
RAF
MEK
ERK1/2
JAK
STAT
ELK
CREBBP
Nucleus
mRNA translation
1. FLT3 Inhibitors
- Target ITD and/or TKD mutations, i.e. Midostaurin and Ponatinib
2. PI3K inhibitors
- BKM120, GDC-0941, BEZ235*
3. mTOR inhibitors
- MK8669, BEZ235*
4. RAF inhibitors
- Sorafenib, LGX818
5. MEK inhibitors
- Selumetinib, PD0325901, MEK162

Supplementary Figure 7: The RAS/RAF/ERK and PI3K/AKT/mTOR pathways – a complex network. FLT3 receptor (blue) dimerisation and anchorage at the cell membrane encourages FLT3 ligand (dark red) binding, which leads to auto-phosphorylation of the receptor (‘P’ hexagon symbol), and activation of the RAS/RAF/MEK (green) and PI3K/AKT/mTOR (red) pathways. Mutant FLT3 receptor can directly activate STAT5, usually regulated by the cytokine receptor/JAK/TYK2 pathway.[1] RAS activity is regulated by NF1, and the active state is represented with the yellow explosion symbol. Some genes can co-activate components of both pathways, i.e. RAS can activate RAF and PI3K, and inhibition of one pathway may therefore lead to activation of the other.[2] Furthermore, mechanisms of drug resistance can evolve in a sample treated with one form of inhibitor, though the acquisition of mutations in genes involved in the alternative pathway.[3] The administration of multiple inhibitors that target a number of key cell-signalling pathways, or in combination with chemotherapeutic agents, may overcome the issue of pathway co-activation or drug resistance. Mutations were detected in *FLT3*, *SHP2* (*PTPN11*), *GRP3* (*RASGRP3*), *RAS*, *NF1*, and *BRAF* (highlighted by the red lightning bolt) in our iAMP21-ALL cohort. Several components of the RAS/RAF/MEK and PI3K/AKT/mTOR pathways are the current focus of novel targeted therapeutic development; examples of targetable genes (numbered stars) and developed therapies are shown in the white box (http://www.mycancergenome.org/content/other/molecular-medicine/anticancer-agents/). *Therapeutic agents that exhibit multi-gene inhibition.

References:

1. Choudhary C, Brandts C, Schwable J, Tickenbrock L, Sargin B, Ueker A, et al. Activation mechanisms of STAT5 by oncogenic Flt3-ITD. Blood. 2007 Jul 1;110(1):370-4.
2. Steelman LS, Chappell WH, Abrams SL, Kempf RC, Long J, Laidler P, et al. Roles of the Raf/MEK/ERK and PI3K/PTEN/Akt/mTOR pathways in controlling growth and sensitivity to therapy-implications for cancer and aging. Aging. 2011 Mar;3(3):192-222.
3. Dail M, Wong J, Lawrence J, O'Connor D, Nakitandwe J, Chen SC, et al. Loss of oncogenic Notch1 with resistance to a PI3K inhibitor in T-cell leukaemia. Nature. 2014 Sep 25;513(7519):512-6.

Patient No.	Unique patient number (UPN)	Patient No. in International iAMP21 Study[1]	Primary chromosomal abnormality	Disease stage	Whole Exome Sequencing	SNP 6.0 array	MLPA - P335-IKZF1-A1 kit	NRAS Sequencing	KRAS Sequencing	BRAF Sequencing	PTPN11 Sequencing	FLT3 Sequencing	NF1 Sequencing	Illumina MiSeq - validation and selective-exon sequencing (Screen B)
1	23229	437	iAMP21	Diagnosis	Y	Y	Y	454	454	454	454	454	454	Y
1b	23229			Remission	Y	Y								Y
2	11005	416	iAMP21	Diagnosis		Y	Y	454	454	454	454	454	454	Y
2b	11005			Remission		Y								Y
3	11564	421	iAMP21	Diagnosis	Y	Y	Y	454	454	454	454	454	454	Y
3b	11564			Remission	Y	Y								Y
4	22129	432	iAMP21	Diagnosis		Y	Y	454	454	454	454	454	454	Y
4b	22129			Remission		Y								Y
5	24207	446	iAMP21	Diagnosis	Y	Y	Y	454	454	454	454	454	454	Y
5b	24207			Remission	Y	Y								Y
6	2904	451	iAMP21	Diagnosis		Y	Y	454	454	454	454	454	454	Y
6b	2904		iAMP21	Relapse			Y							Y
7	24259	447	iAMP21	Diagnosis	Y	Y	Y	454	454	454	454	454	454	Y
7b	24259			Remission	Y	Y								Y
8	22007	431	iAMP21	Diagnosis		Y	Y	454	454	454	454	454	454	Y
8b	22007			Remission		Y								Y
9	5898	481	iAMP21	Diagnosis	Y	Y	Y	454	454	454	454	454	454	Y
9b	5898			Remission	Y	Y								
10	3382	453	iAMP21	Diagnosis		Y	Y	454	454	454	454	454	454	Y
11	22340	434	iAMP21	Diagnosis		Y	Y	454	454	454	454	454		Y
11b	22340			Remission		Y								Y
12	8743	505	iAMP21	Diagnosis		Y	Y	454	454	454	454	454		Y
12b	8743		iAMP21	Relapse		Y	Y	454	454	454	454	454		Y
13	12085	424	iAMP21	Diagnosis		Y	Y	454	454	454	454	454		Y
13b	12085			Remission		Y								Y
14	21567	430	iAMP21	Diagnosis		Y	Y	454	454	454	IM	454		Y
14b	21567			Remission		Y								
15	22322	433	iAMP21	Diagnosis		Y	Y	454	454	454	454	454		Y
15b	22322			Remission		Y								
16	20724	429	iAMP21	Diagnosis		Y	Y	454	454	454	454	454		
16b	20724			Remission		Y								
17	6783	486	iAMP21	Diagnosis		Y	Y	454	454	454	454	454		Y
18	9864	512	iAMP21	Diagnosis		Y	Y	454	454	454	454	454		Y
19	3743	456	iAMP21	Diagnosis		Y	Y	454	454	454	454	454		Y
20	5674	477	iAMP21	Diagnosis		Y	Y	454	454	454	454	454		Y
21	23982	444	iAMP21	Diagnosis	Y	Y		454	454	454	454	454	454	Y
21b	23982			Remission	Y	Y								Y
22	25452	521	iAMP21	Diagnosis		Y		IM	IM	IM	IM	IM		Y
23	25453	522	iAMP21	Diagnosis		Y		IM	IM	IM	IM	IM		Y
24	25454	523	iAMP21	Diagnosis		Y		IM	IM	IM	IM	IM		Y
24b	25454		iAMP21	Relapse		Y		IM	IM	IM	IM	IM		Y
25	25455	524	iAMP21	Diagnosis		Y		IM	IM	IM	IM	IM		Y
26	24655	195	iAMP21	Diagnosis		Y		454	454	454	454	454		
26b	24655			Remission		Y								
27	23765	442	iAMP21	Diagnosis			Y	454	454	454	454	454	454	Y
27b	23765			Remission			Y							
28	9028	511	iAMP21	Diagnosis			Y	454	454	454	454	454	454	Y
28b	9028			Remission				454	454	454	454	454	454	Y
29	24447	320	iAMP21	Diagnosis			Y	454	454	454	454	454	454	Y
30	24452	370	iAMP21	Diagnosis			Y	454	454	454	IM	454	454	Y
31	6008	482	iAMP21	Diagnosis			Y	454	454	454	454	454	454	Y
32	11061	417	iAMP21	Diagnosis			Y	454	454	454	454	454	454	Y
33	4134	461	iAMP21	Diagnosis			Y	454	454	454	454	454		Y
33b	4134		iAMP21	Relapse			Y	454	454	454	454	454	454	Y
34	4279	465	iAMP21	Diagnosis			Y	454	454	454	454	454		Y
34b	4279		iAMP21	Relapse			Y	454	454	454	454	454	454	Y
35	4780	473	iAMP21	Diagnosis			Y	454	454	454	IM	454		Y
35b	4780		iAMP21	Relapse			Y	454	454	454	454	454	454	
36	4405	467	iAMP21	Diagnosis			Y	454	454	454	454	IM		Y
36b	4405		iAMP21	Relapse			Y							Y
37	7828	502	iAMP21	Diagnosis			Y	454	454	454	454	454		Y
38	11158	418	iAMP21	Diagnosis			Y	454	454	454	454	454		Y
39	11557	420	iAMP21	Diagnosis			Y	454	454	454	454	454		Y
40	6996	491	iAMP21	Diagnosis			Y	454	454	454	454	454		Y
41	6788	487	iAMP21	Diagnosis			Y	454	454	454	IM	454		F
42	24454	372	iAMP21	Diagnosis			Y	454	454	454	454	454		
43	23299	439	iAMP21	Diagnosis	Y	Y	Y							
43b	23299			Remission	Y	Y								
44	23888	444	iAMP21	Diagnosis	Y	Y	Y							
44b	23888			Remission	Y	Y								
45*	19578	426	iAMP21	Diagnosis				IM	IM		IM	IM		
46b*	23317		iAMP21	Relapse				IM	IM		IM	IM		
47	8947		B-other	Diagnosis				Y	Y			Y		
48	9146		B-other	Diagnosis				Y	Y			Y		
49	9465		B-other	Diagnosis				Y	Y			Y		
50	9525		B-other	Diagnosis				Y	Y			Y		
51	9859		B-other	Diagnosis				Y	Y			Y		
52	9877		B-other	Diagnosis				Y	Y			Y		
53	10054		B-other	Diagnosis				Y	Y			Y		
54	10077		B-other	Diagnosis				Y	Y			Y		
55	10173		B-other	Diagnosis				Y	Y			Y		
56	10184		B-other	Diagnosis				Y	Y			Y		
57	10186		B-other	Diagnosis				Y	Y			Y		
58	10297		B-other	Diagnosis				Y	Y			Y		
59	10419		B-other	Diagnosis				Y	Y			Y		
60	10442		B-other	Diagnosis				Y	Y			Y		
61	10656		B-other	Diagnosis				Y	Y			Y		
62	10743		B-other	Diagnosis				Y	Y			Y		
63	10817		B-other	Diagnosis				Y	Y			Y		
64	11104		B-other	Diagnosis				Y	Y			Y		
65	11111		B-other	Diagnosis				Y	Y			Y		
66	11390		B-other	Diagnosis				Y	Y			Y		
67	11832		B-other	Diagnosis				Y	Y			Y		
68	11889		B-other	Diagnosis				Y	Y			Y		
69	11957		B-other	Diagnosis				Y	Y			Y		
70	12021		B-other	Diagnosis				Y	Y			Y		
71	12286		B-other	Diagnosis				Y	Y			Y		
72	12324		B-other	Diagnosis				Y	Y			Y		
73	12476		B-other	Diagnosis				Y	Y			Y		
74	12634		B-other	Diagnosis				Y	Y			Y		
75	20323		B-other	Diagnosis				Y	Y			Y		
76	20515		B-other	Diagnosis				Y	Y			Y		
77	20548		B-other	Diagnosis				Y	Y			Y		
78	20574		B-other	Diagnosis				Y	Y			Y		
79	20683		B-other	Diagnosis				Y	Y			Y		
80	20709		B-other	Diagnosis				Y	Y			Y		
81	20716		B-other	Diagnosis				Y	Y			Y		
82	20720		B-other	Diagnosis				Y	Y			Y		
83	20748		B-other	Diagnosis				Y	Y			Y		
84	20750		B-other	Diagnosis				Y	Y			Y		
85	20753		B-other	Diagnosis				Y	Y			Y		
86	20759		B-other	Diagnosis				Y	Y			Y		
87	20764		B-other	Diagnosis				Y	Y			Y		
88	20874		B-other	Diagnosis				Y	Y			Y		
89	21198		B-other	Diagnosis				Y	Y			Y		
90	21207		B-other	Diagnosis				Y	Y			Y		
91	21208		B-other	Diagnosis				Y	Y			Y		
92	22194		B-other	Diagnosis				Y	Y			Y		
93	22387		B-other	Diagnosis				Y	Y			Y		
94	22388		B-other	Diagnosis				Y	Y			Y		
95	22408		B-other	Diagnosis				Y	Y			Y		
96	22499		B-other	Diagnosis				Y	Y			Y		
97	22572		B-other	Diagnosis				Y	Y			Y		
98	22584		B-other	Diagnosis				Y	Y			Y		
99	22673		B-other	Diagnosis				Y	Y			Y		
100	22689		B-other	Diagnosis				Y	Y			Y		
101	22790		B-other	Diagnosis				Y	Y			Y		
102	22861		B-other	Diagnosis				Y	Y			Y		
103	22889		B-other	Diagnosis				Y	Y			Y		
104	22918		B-other	Diagnosis				Y	Y			Y		
105	22936		B-other	Diagnosis				Y	Y			Y		
106	22964		B-other	Diagnosis				Y	Y			Y		
107	22972		B-other	Diagnosis				Y	Y			Y		
108	22980		B-other	Diagnosis				Y	Y			Y		
109	23065		B-other	Diagnosis				Y	Y			Y		
110	23075		B-other	Diagnosis				Y	Y			Y		
111	23114		B-other	Diagnosis				Y	Y			Y		
112	20399		B-other	Diagnosis				Y	Y			Y		
113	8768		HeH	Diagnosis				Y	Y			Y		
114	9154		HeH	Diagnosis				Y	Y			Y		
115	10069		HeH	Diagnosis				Y	Y			Y		
116	10247		HeH	Diagnosis				Y	Y			Y		
117	10254		HeH	Diagnosis				Y	Y			Y		
118	10400		HeH	Diagnosis				Y	Y			Y		
119	10410		HeH	Diagnosis				Y	Y			Y		

120	10576		HeH	Diagnosis				Y	Y			Y		
121	10626		HeH	Diagnosis				Y	Y			Y		
122	10631		HeH	Diagnosis				Y	Y			Y		
123	10720		HeH	Diagnosis				Y	Y			Y		
124	10740		HeH	Diagnosis				Y	Y			Y		
125	10837		HeH	Diagnosis				Y	Y			Y		
126	10996		HeH	Diagnosis				Y	Y			Y		
127	11103		HeH	Diagnosis				Y	Y			Y		
128	11154		HeH	Diagnosis				Y	Y			Y		
129	11301		HeH	Diagnosis				Y	Y			Y		
130	11407		HeH	Diagnosis				Y	Y			Y		
131	11570		HeH	Diagnosis				Y	Y			Y		
132	11635		HeH	Diagnosis				Y	Y			Y		
133	11674		HeH	Diagnosis				Y	Y			Y		
134	11735		HeH	Diagnosis				Y	Y			Y		
135	11884		HeH	Diagnosis				Y	Y			Y		
136	12325		HeH	Diagnosis				Y	Y			Y		
137	12376		HeH	Diagnosis				Y	Y			Y		
138	12628		HeH	Diagnosis				Y	Y			Y		
139	12728		HeH	Diagnosis				Y	Y			Y		
140	12747		HeH	Diagnosis				Y	Y			Y		
141	19646		HeH	Diagnosis				Y	Y			Y		
142	19764		HeH	Diagnosis				Y	Y			Y		
143	19994		HeH	Diagnosis				Y	Y			Y		
144	20384		HeH	Diagnosis				Y	Y			Y		
145	20712		HeH	Diagnosis				Y	Y			Y		
146	20781		HeH	Diagnosis				Y	Y			Y		
147	21346		HeH	Diagnosis				Y	Y			Y		
148	21635		HeH	Diagnosis				Y	Y			Y		
149	21750		HeH	Diagnosis				Y	Y			Y		
150	21998		HeH	Diagnosis				Y	Y			Y		
151	22147		HeH	Diagnosis				Y	Y			Y		
152	22238		HeH	Diagnosis				Y	Y			Y		
153	22341		HeH	Diagnosis				Y	Y			Y		
154	22420		HeH	Diagnosis				Y	Y			Y		
155	22434		HeH	Diagnosis				Y	Y			Y		
156	22468		HeH	Diagnosis				Y	Y			Y		
157	22676		HeH	Diagnosis				Y	Y			Y		
158	22799		HeH	Diagnosis				Y	Y			Y		
159	22928		HeH	Diagnosis				Y	Y			Y		
160	23031		HeH	Diagnosis				Y	Y			Y		

Supplementary Table 1: A summary of genetic studies per patient sample used in this study. Studies were approved by the relevant institutional ethics committee(s) and written informed consent was obtained for each patient from parents, legal guardians or themselves. The cohort was comprised of 46 samples from patients with iAMP21-ALL. Cases 1-44 were derived from patient material which was used to investigate the incidence of RAS mutations in iAMP21-ALL. *Patients 45 and 46b refers to the xenograft material of the patient sample; viable cells were used as a control to assess the effect of selumetinib in iAMP21-ALL (Supplementary Figure 6). Matched remission and /or relapse material was available for select iAMP21-ALL cases (highlighted in grey). Clinical and cytogenetic data is available from our previous studies.[1,2] iAMP21-ALL was confirmed in each case through the acquisition of multiple *RUNX1* signals (relative to *ETV6*) on interphase cells or metaphase spreads by FISH. 66 (patients 47-112) and 48 (patients 113-160) diagnostic patient samples with no recurrent chromosomal abnormality (defined as B-other) or high hyperdipoidy (HeH), respectively, were used in this study. Genetic studies performed in this study are highlighted in yellow and included SNP6.0 array, MLPA analysis (P335-IKZF1 kit) and targeted sequencing (using Roche 454 or Illumina MiSeq technology). Abbreviations: Y, analysis performed; 454, Roche 454 sequencing technology; IM, Illumina MiSeq sequencing; F, failed.

References

1 Harrison CJ, Moorman AV, Schwab C, Carroll AJ, Raetz EA, Devidas M, et al. An international study of intrachromosomal amplification of chromosome 21 (iAMP21): cytogenetic characterization and outcome. Leukemia : official journal of the Leukemia Society of America, Leukemia Research Fund, UK. 2014 May;28(5):1015-21.
2 Rand V, Parker H, Russell LJ, Schwab C, Ensor H, Irving J, et al. Genomic characterization implicates iAMP21 as a likely primary genetic event in childhood B-cell precursor acute lymphoblastic leukemia. Blood. 2011 Jun 23;117(25):6848-55.

Patient ID	Disease stage	SNP 6.0 array	NRAS chr1:115247084-115259515	SOS1 chr2:39208689-39347604	RAF1 chr3:12625099-12705700	BRAF chr7:140433812-140624564	HRAS chr11:532241-535550	CBL chr11:119076985-119178859	KRAS chr12:25358179-25403854	PTPN11 chr12:112856535-112924727	FLT3 chr13:28577410-28674729	MAP2K1 chr15:66679210-66783882	MAPK3 chr16:30125425-30134630	CRK chr17:1324646-1359561	NF1 chr17:29421944-29704695	GRB2 chr17:73314156-73401790	MAP2K2 chr19:4090319-4124126	MAPK1 chr22:22123318-22221970	Copy number abnormality (CNA) information
1	Diagnosis	Y	2	2	2	1	2	2	2	2	2	2	2	2	2	2	2	2	*BRAF* loss, chr7:138489883-141615098, CN=1
1b	Remission	Y	2	2	2	2	2	2	2	2	2	2	2	2	2	2	2	2	
2	Diagnosis	Y	2	2	2	2	2	2	2	2	2	2	2	2	2	2	2	3	*MAPK1* gain, chr22, CN=3
2b	Remission	Y	2	2	2	2	2	2	2	2	2	2	2	2	2	2	2	2	
3	Diagnosis	Y	2	2	2	2	2	2	2	2	2	2	2	2	2	2	2	2	
3b	Remission	Y	2	2	2	2	2	2	2	2	2	2	2	2	2	2	2	2	
4	Diagnosis	Y	2	2	2	2	2	2	2	2	2	2	2	2	2	2	2	2	
4b	Remission	Y	2	2	2	2	2	2	2	2	2	2	2	2	2	2	2	2	
5	Diagnosis	Y	2	2	2	2	2	2	2	2	2	2	2	2	2	2	2	2	
5b	Remission	Y	2	2	2	2	2	2	2	2	2	2	2	2	2	2	2	2	
6	Diagnosis	Y	2	2	2	1	2	2	2	2	2	2	2	2	2	2	2	2	*BRAF* loss, chr7:123075091-159117317, CN=1
6b	Relapse																		
7	Diagnosis	Y	2	2	2	2	2	2	2	2	2	2	2	2	2	2	2	2	
7b	Remission	Y	2	2	2	2	2	2	2	2	2	2	2	2	2	2	2	2	
8	Diagnosis	Y	2	2	2	2	2	2	2	2	2	2	2	2	2	2	2	2	
8b	Remission	Y	2	2	2	2	2	2	2	2	2	2	2	2	2	2	2	2	
9	Diagnosis	Y	2	2	2	2	2	2	2	2	3	2	2	1	3	3	2	2	*FLT3* gain chr13:28577688-28653242, CN=3<br>*NF1* and *GRB2* gain, chr17:21524160-81049726, CN=3<br>*CRK* loss, chr17:21524160-81049726, CN=1
9b	Remission	Y	2	2	2	2	2	2	2	2	2	2	2	2	2	2	2	2	
10	Diagnosis	Y	2	2	2	2	2	1	2	2	2	2	2	2	2	3	2	2	*CBL* loss, chr11:101085126-128054642, CN=1<br>*GRB2* gain, chr17:40024271-80907559, CN=3
11	Diagnosis	Y	2	2	2	2	2	2	2	2	2	2	2	2	2	2	2	2	
11b	Remission	Y	2	2	2	2	2	2	2	2	2	2	2	2	2	2	2	2	
12	Diagnosis	Y	2	2	2	2	2	1	2	2	2	2	2	2	2	2	2	2	*CBL* loss, chr11:96439395-134944770, CN=1
12b	Relapse	Y	2	2	2	2	2	1	2	2	2	2	2	2	2	2	2	2	*CBL* loss, chr11:96439395-134944770, CN=1
13	Diagnosis	Y	2	2	2	2	2	2	2	2	2	2	2	2	1	2	2	2	*NF1* loss chr17:27671072-30298842, CN=1
13b	Remission	Y	2	2	2	2	2	2	2	2	2	2	2	2	2	2	2	2	
14	Diagnosis	Y	2	2	2	2	2	2	2	2	2	2	2	3	3	3	2	2	*NF1* , GRB2 and CRK gain, chr17, CN=3
14b	Remission	Y	2	2	2	2	2	2	2	2	2	2	2	2	2	2	2	2	
15	Diagnosis	Y	2	2	2	2	2	1	2	2	2	2	2	2	2	2	2	2	*CBL* loss chr11:103700452-122725504, CN=1
15b	Remission	Y	2	2	2	2	2	2	2	2	2	2	2	2	2	2	2	2	
16	Diagnosis	Y	2	2	2	2	2	2	2	2	2	2	2	2	2	2	2	2	
16b	Remission	Y	2	2	2	2	2	2	2	2	2	2	2	2	2	2	2	2	
17	Diagnosis	Y	2	2	2	2	2	2	2	2	2	4	2	2	2	2	2	2	*MAP2K1* gain chr15:60309674-68683575, CN=4
18	Diagnosis	Y	2	2	2	2	2	2	2	2	2	2	2	2	2	2	2	2	
19	Diagnosis	Y	2	2	2	2	2	1	2	2	2	2	2	2	2	2	2	2	*CBL* loss, chr11:78035832-134944770, CN=1
20	Diagnosis	Y	2	2	2	2	2	2	2	2	2	2	2	2	2	2	2	2	
21	Diagnosis	Y	2	2	2	2	2	2	2	2	2	2	2	2	2	2	2	2	
21b	Remission	Y	2	2	2	2	2	2	2	2	2	2	2	2	2	2	2	2	
22	Diagnosis	Y	2	2	2	2	2	2	2	2	3	2	2	2	2	2	2	2	*FLT3* gain, chr13:21197493-46080684, CN=3
23	Diagnosis	Y	2	2	2	2	2	2	2	2	2	2	2	2	2	2	2	2	
24	Diagnosis	Y	2	2	2	1	2	2	2	2	2	2	2	2	2	2	2	2	*BRAF* loss chr7:120150796-159119708, CN=1
24b	Relapse	Y	2	2	2	1	2	2	2	2	2	2	2	2	2	2	2	2	*BRAF* loss chr7:120150796-159119708, CN=1
25	Diagnosis	Y	2	2	2	2	2	2	2	2	2	2	2	2	2	2	2	2	
26	Diagnosis	Y	2	2	2	2	2	2	2	2	2	2	2	2	2	2	2	2	
26b	Remission	Y	2	2	2	2	2	2	2	2	2	2	2	2	2	2	2	2	
43	Diagnosis	Y	2	2	2	2	2	1	2	2	2	3	2	1	2	2	2	2	*CBL* loss chr11:89850834-134944770, CN=1<br>*MAP2K1* gain chr15:62606091-71815395, CN=3<br>*CRK* loss chr17:0-16571504, CN=1
43b	Remission	Y	2	2	2	2	2	2	2	2	2	2	2	2	2	2	2	2	
44	Diagnosis	Y	2	2	2	2	2	2	2	2	2	2	2	2	2	2	2	2	
44b	Remission	Y	2	2	2	2	2	2	2	2	2	2	2	2	2	2	2	2	

Supplementary Table 2: Copy number status of RAS pathway genes in iAMP21-ALL. SNP6.0 copy number profiling was performed on 28 diagnostic and 2 relapse iAMP21-ALL patient samples. Matched remission material was available for 17 cases. Arrays were prepared by The Paterson Institute Microarray Service (Manchester, UK) or AROS Applied Biotechnology AS (Aarhus, Denmark). Copy number and segmentation analysis was performed using Genotyping Console version 4.1.4 (Affymetrix, Santa Clara, California, USA). Regions of genomic gain and loss were confirmed by manual assessment. Matched remission samples facilitated the exclusion of germline copy number variants (CNV) and were used to eliminate potential germline variants in unmatched diagnostic samples, in addition to the list of CNV reported in the Toronto Database of Genomic Variants (DGV). Regions of CNA were mapped in relation to the Genome Reference Consortium Human genome build 37 (GRCh37). The copy number status of 16 genes that represent components of the RAS pathway is shown. Most deletions formed part of a larger abnormality (T) which was often visible by karyotype analysis.

Supplementary Table 3: Primer sequences used to screen and validate RAS pathway mutations. A) Primers were used to screen for RAS pathway mutations using the Roche 454 FLX and / or Illumina MiSeq. RAS pathway primers were composed of the consensus sequence (red text) for binding of the Access Array Barcode Library primers and the target-specific sequence (black text). *NRAS* , *KRAS* , *BRAF* , and *NF1* primers have been described previously.1-3 Primers targeting *NRAS* exon 2 and *FLT3* exon 16 were used to validate putative mutations identified by whole exome sequencing using Sanger sequencing. B) Primers used to amplify *FLT3* transcripts that harbour ITD mutations, designed to bind to exons 13 (forward primer) and 16 (reverse primer). CS1 - tag, consensus sequence 1; CS2 - tag, consensus sequence 2.

A) Sequencing Primers for RAS Pathway Analysis

Gene Name	Chromosome	Exon #	Forward primer sequence (+ CS1 - tag)	Reverse primer sequence (+ CS2 - tag)	Illumina MiSeq Sequencing Primers for RAS Pathway Analysis (if different): Forward primer sequence (+ CS1 - tag)	Reverse primer sequence (+ CS2 - tag)
KRAS	1	2	ACACTGACGACATGGTTCTACACGTCTGCAGTCAACTGGAAT	TACGGTAGCAGAGACTTGGTCTAGAATGGTCCTGCACCAGTAA		
KRAS	1	3	ACACTGACGACATGGTTCTACATGTAATAATCCAGACTGTGTTTCTCC	TACGGTAGCAGAGACTTGGTCTCAGGGATATTACCTACCTCATAAACA		
NRAS	12	2	ACACTGACGACATGGTTCTACAGGCCGATATTAATCCGGTGT	TACGGTAGCAGAGACTTGGTCTTCCGACAAGTGAGAGACAGG		
NRAS	12	3	ACACTGACGACATGGTTCTACACCCTCCCTGCCCCCTTAC	TACGGTAGCAGAGACTTGGTCTCAATGTCAAACAACCTAAAACCA		
BRAF	11	11	ACACTGACGACATGGTTCTACATCTTCCTGTATCCCTCTCAGG	TACGGTAGCAGAGACTTGGTCTAGTTTATTGATGCGAACAGTGA		
BRAF	11	15	ACACTGACGACATGGTTCTACATTGACTCTAAGAGGAAAGATGAAG	TACGGTAGCAGAGACTTGGTCTGTAACTCAGCAGCATCTCAGG		
PTPN11	12	2	ACACTGACGACATGGTTCTACAGGGACAGGGAAGGTCTTGAT	TACGGTAGCAGAGACTTGGTCTAAATGCAGGCAGCAAGCTAT		
PTPN11	12	3	ACACTGACGACATGGTTCTACACGTTCCTTGGGTTTCTTTCA	TACGGTAGCAGAGACTTGGTCTCACAAGGGAGCAGCAGACTT		
PTPN11	12	4	ACACTGACGACATGGTTCTACATTGATCAATCCCTTGGAGGA	TACGGTAGCAGAGACTTGGTCTACATCTTGCCAGACCCATTT		
PTPN11	12	5	ACACTGACGACATGGTTCTACAGAGAGTGCTTGAAAACACTAATGTAA	TACGGTAGCAGAGACTTGGTCTTGATAGTTGAAGCTGCAATGG		
PTPN11	12	6	ACACTGACGACATGGTTCTACACCTCTGTCCGTGCCTTTATG	TACGGTAGCAGAGACTTGGTCTGTGTCAATCAATGGCCGAAT		
PTPN11	12	7	ACACTGACGACATGGTTCTACAGCCTGACCCAGATGAACATT	TACGGTAGCAGAGACTTGGTCTCCGATGTGCTAACAAGAGCA		
PTPN11	12	8	ACACTGACGACATGGTTCTACACATCAGGCAGTGTTCACGTT	TACGGTAGCAGAGACTTGGTCTCTTTCAGGACATGAGGAAGGA		
PTPN11	12	9	ACACTGACGACATGGTTCTACATCATCATGGTAAGCTTTGCTTT	TACGGTAGCAGAGACTTGGTCTTTCCTAAACATGGCCAATCTG		
PTPN11	12	10	ACACTGACGACATGGTTCTACAAGATGCGAAACAGGCCATT	TACGGTAGCAGAGACTTGGTCTGAGTAACGGCAAGACCCTGA		
PTPN11	12	11.1	ACACTGACGACATGGTTCTACATAGAACCGGGTGATTCCTCA	TACGGTAGCAGAGACTTGGTCTTGCAGTTGCTCTATGCCTCA		
PTPN11	12	11.2	ACACTGACGACATGGTTCTACACAGCCTGTCCCTGTCTCCTA	TACGGTAGCAGAGACTTGGTCTCCCACCACCTCAATACCTACA		
PTPN11	12	12	ACACTGACGACATGGTTCTACACAGGCACGTTGTACTGGAGA	TACGGTAGCAGAGACTTGGTCTCGTGAGCACTTTCCTTCCAT		
PTPN11	12	13	ACACTGACGACATGGTTCTACACTGTAGCCATTGCAACATGC	TACGGTAGCAGAGACTTGGTCTCCAAGAGGCCTAGCAAGAGA		
PTPN11	12	14	ACACTGACGACATGGTTCTACACCCTCACATGTGCACTCTTC	TACGGTAGCAGAGACTTGGTCTCCTGGCCAGCTTAGGAAA		
PTPN11	12	15	ACACTGACGACATGGTTCTACATCTCTCCCTGGGAATGTCAG	TACGGTAGCAGAGACTTGGTCTCCAAATTGCTTGCCTGCTTA		
FLT3	13	2	ACACTGACGACATGGTTCTACATGGTTCACAGGAAGACAGCA	TACGGTAGCAGAGACTTGGTCTTGGTAGAGGACTTGCCACAA		
FLT3	13	3	ACACTGACGACATGGTTCTACATTTGGTCATGCATTTGGAAG	TACGGTAGCAGAGACTTGGTCTAGCGTGAACCGTGACTTCTT		
FLT3	13	4	ACACTGACGACATGGTTCTACAGCACCGAGAGAGCAGAGG	TACGGTAGCAGAGACTTGGTCTCCTTTCACGTGAGGAATGTG		
FLT3	13	5	ACACTGACGACATGGTTCTACAGCAGACTGCTGTGAGGGTTT	TACGGTAGCAGAGACTTGGTCTTCCATCACCGGTACCTCCTA		
FLT3	13	6	ACACTGACGACATGGTTCTACATTTGGCAAAAGTGAAGACTGG	TACGGTAGCAGAGACTTGGTCTTGGCTTGAATAACCTTCCCATA		
FLT3	13	7	ACACTGACGACATGGTTCTACAAAAACTTTTGCATTCATTTCCATT	TACGGTAGCAGAGACTTGGTCTAGACTGGCAAGCCCAGTTAC		
FLT3	13	8	ACACTGACGACATGGTTCTACAACGGTGCCTTTAGAAACTGG	TACGGTAGCAGAGACTTGGTCTCAGAGAACCAAGCCCTCCTA		
FLT3	13	9	ACACTGACGACATGGTTCTACACATGCCTGGCTTCTCTCATAA	TACGGTAGCAGAGACTTGGTCTGCAAACATCCTCTTTGTCATCA		
FLT3	13	10	ACACTGACGACATGGTTCTACATGCATCTTTGTTGCTGTCCT	TACGGTAGCAGAGACTTGGTCTGTGACCGGCTCCTCAGATAA		
FLT3	13	11	ACACTGACGACATGGTTCTACAGCAACCTGGATTGAGACTCC	TACGGTAGCAGAGACTTGGTCTTCTGCAGAACTGCCTATTCCT		
FLT3	13	12	ACACTGACGACATGGTTCTACACTCTTGGAAACTCCCATTTGA	TACGGTAGCAGAGACTTGGTCTTCTGTTTCATCGCTGAGTGAC		
FLT3	13	13	ACACTGACGACATGGTTCTACATCACACACTGACCCTATACTCTCC	TACGGTAGCAGAGACTTGGTCTTTCCAATCTGATGACAGCTCAC		
FLT3	13	14	ACACTGACGACATGGTTCTACAGGATTTCTGCAGCGAGTTCT	TACGGTAGCAGAGACTTGGTCTCCCAGCCAGTGAGCTTATTT		
FLT3	13	15	ACACTGACGACATGGTTCTACATTGTAGTAGCCCTTCTAAGCCATT	TACGGTAGCAGAGACTTGGTCTCCAGTGAACTTTAGCAAGTGAGG		
FLT3	13	16	ACACTGACGACATGGTTCTACAAACTCATAAGTGAATTTGCTTCCA	TACGGTAGCAGAGACTTGGTCTTCTTCTCAATACGTTTCCCTATGA		
FLT3	13	17	ACACTGACGACATGGTTCTACACACATTCCACACTACAGCGACT	TACGGTAGCAGAGACTTGGTCTGGCTCACCTGGGAATTAGAA		
FLT3	13	18	ACACTGACGACATGGTTCTACATCTCAAAGTAGTTGCCCTAGGTTT	TACGGTAGCAGAGACTTGGTCTAAAGTCTTTGTTCAGAATGTTCAGG		
FLT3	13	19	ACACTGACGACATGGTTCTACAGCTAAGAACCGGTCACTGAAA	TACGGTAGCAGAGACTTGGTCTTCTGAGCTCTTAGCTGAAACCA		
FLT3	13	20	ACACTGACGACATGGTTCTACAGCAGCTACCATGGATGTGTC	TACGGTAGCAGAGACTTGGTCTGACACTGCAGCCACAGCTAA		
FLT3	13	21	ACACTGACGACATGGTTCTACACTGGTCAAGCTAACGGGTTC	TACGGTAGCAGAGACTTGGTCTGGCACATGACAGAAGCTGTG		
FLT3	13	22	ACACTGACGACATGGTTCTACATGTCAAGAAGGTATTCCTCCACT	TACGGTAGCAGAGACTTGGTCTTCCAGCTTGTGTGGGTGTT		
FLT3	13	23	ACACTGACGACATGGTTCTACATTTAGCCATGGTAGGCTTCAA	TACGGTAGCAGAGACTTGGTCTAGTGGCCAATTTCCTCTGATT		
FLT3	13	24	ACACTGACGACATGGTTCTACAAGGCAAGTAGTGGGCGAGT	TACGGTAGCAGAGACTTGGTCTGCATCCTTAAGAGAGCCACCT		
NF1	17	2	ACACTGACGACATGGTTCTACATTGGAGGTTGATTGAAAATCG	TACGGTAGCAGAGACTTGGTCTAGCAAATTCCCCAAAACACA		
NF1	17	3	ACACTGACGACATGGTTCTACATGCCATTTCTGTTTGCCTTAG	TACGGTAGCAGAGACTTGGTCTTTCTCAAGATCAAATTCACAAAGC		
NF1	17	4	ACACTGACGACATGGTTCTACATCATTATTTTTGTTCTGTGTGTGTG	TACGGTAGCAGAGACTTGGTCTTTCATATTACTTCAGTAGTCCCATGT		
NF1	17	5	ACACTGACGACATGGTTCTACAAGAGATGATGTCTTGCTATGTTGC	TACGGTAGCAGAGACTTGGTCTTTCATGATACTAGTTTTTGACCCAGT		
NF1	17	6	ACACTGACGACATGGTTCTACATGTCCAAGGCATATTTGCTG	TACGGTAGCAGAGACTTGGTCTTGATTCAGGATGCTAACAACAG		
NF1	17	7	ACACTGACGACATGGTTCTACAGCCTGGAAAAGGTAAGTTACAA	TACGGTAGCAGAGACTTGGTCTAAAAAAAAAAAATCAATCGTATCC		
NF1	17	8	ACACTGACGACATGGTTCTACATTTTAAAAATGTTGCCCTTGG	TACGGTAGCAGAGACTTGGTCTAAGCCTAAAGTAATACACACCTTGA		
NF1	17	9	ACACTGACGACATGGTTCTACAGCTACATCTGGAATAGAAGAAACTTC	TACGGTAGCAGAGACTTGGTCTCGAAGAGTCAGAACTTTAATGTTAGC		
NF1	17	10	ACACTGACGACATGGTTCTACATGCCATTTGTGTGGGTAATG	TACGGTAGCAGAGACTTGGTCTACGAACATCAATACATAATAGTGAGA		
NF1	17	11	ACACTGACGACATGGTTCTACAAATCTGCTTTTTTTTTTCTTTTTC	TACGGTAGCAGAGACTTGGTCTTTCCAAAGCATCTTATTAACTCCA		
NF1	17	12	ACACTGACGACATGGTTCTACAGACGTTGTTTGAAGACTTTGGA	TACGGTAGCAGAGACTTGGTCTTGCAATAGAAAGGAGGTGAGA		
NF1	17	13	ACACTGACGACATGGTTCTACAACCTTATGCTTACTATTGAGTGTTTCT	TACGGTAGCAGAGACTTGGTCTCGTTTCAGCTAAACCCAATTAAC		
NF1	17	14	ACACTGACGACATGGTTCTACATCACCCCTAAAAGAAACTTGG	TACGGTAGCAGAGACTTGGTCTTTTCCAGAAATGACATCTACCTG		
NF1	17	15	ACACTGACGACATGGTTCTACATGAAACTACAAATGAAAGAGCTCAA	TACGGTAGCAGAGACTTGGTCTAATACAAAACCATAAAACCTTTGGA		
NF1	17	16	ACACTGACGACATGGTTCTACATGCATTAGGTTATTGATGATGC	TACGGTAGCAGAGACTTGGTCTTTGCCAGTCATTGTCCTCTG		
NF1	17	17	ACACTGACGACATGGTTCTACACTTGGTTGTCAGTGCTTCAG	TACGGTAGCAGAGACTTGGTCTCCACAAAGAGCAGTAATAGAACTAGA		
NF1	17	18	ACACTGACGACATGGTTCTACATTTATACATAAAATTACCCAAGTTGC	TACGGTAGCAGAGACTTGGTCTTGTGCTTTGAGGCAGACTGA		
NF1	17	19	ACACTGACGACATGGTTCTACATCCTGTGAGGTTAGTGAAAGGAA	TACGGTAGCAGAGACTTGGTCTTGCACGTATCTTGGATTTACTTC		
NF1	17	20	ACACTGACGACATGGTTCTACATGTGATCAGGAATAGCTTTTGA	TACGGTAGCAGAGACTTGGTCTTGTTTACTTTACTGAGCGACTCTTG		
NF1	17	21.1	ACACTGACGACATGGTTCTACATGTCATGGAAGAAATGTTGGA	TACGGTAGCAGAGACTTGGTCTCTGCGTTTCCCTCTGAAGAC		
NF1	17	21.2	ACACTGACGACATGGTTCTACAGTGTGCCTCCAGCAGAGAA	TACGGTAGCAGAGACTTGGTCTTTCATAGAGAAAGGTGAAAAATAAGA		
NF1	17	22	ACACTGACGACATGGTTCTACATGGGTGCACTTACTCTGTGTG	TACGGTAGCAGAGACTTGGTCTAAGAATGGCCAGTTATTTTATCAAT		
NF1	17	23	ACACTGACGACATGGTTCTACATGCCTTCTCTTTTGTCTATATCTGA	TACGGTAGCAGAGACTTGGTCTAGGCACAAAGCTTAGGGAAA		
NF1	17	24	ACACTGACGACATGGTTCTACACGTCATGTCACTTAGGTTATCTGG	TACGGTAGCAGAGACTTGGTCTGCATCTTTACATATTTCAAACACAAA		
NF1	17	25	ACACTGACGACATGGTTCTACATGAGGGGAAGTGAAAGAACTTG	TACGGTAGCAGAGACTTGGTCTGCAAAATTTCACTGATCATATTACTT		
NF1	17	26	ACACTGACGACATGGTTCTACACCATTCACACCATGCACATA	TACGGTAGCAGAGACTTGGTCTTGCTTCTCTTACATGCCAGTTC		
NF1	17	27	ACACTGACGACATGGTTCTACACTTCAGCAAGGCCATGTTAGT	TACGGTAGCAGAGACTTGGTCTTATTCTGAAGGATTTGCTATGTGC		
NF1	17	28	ACACTGACGACATGGTTCTACATATTAATCAGTCATCATTTGCCTTA	TACGGTAGCAGAGACTTGGTCTCCTCAGCACACACATACACACA		
NF1	17	29	ACACTGACGACATGGTTCTACAGACTCTCTTCCGAGGCAACA	TACGGTAGCAGAGACTTGGTCTAAACAGCGGTTCTATGTGAAA		
NF1	17	30	ACACTGACGACATGGTTCTACAACGTTGCACTTGGCTTAATG	TACGGTAGCAGAGACTTGGTCTCCACACACCATCAGCAGCTA		
NF1	17	31	ACACTGACGACATGGTTCTACATGCTGTATGTAGTCGGTGCTG	TACGGTAGCAGAGACTTGGTCTAACAAATGCCCACAAATTGC		
NF1	17	32	ACACTGACGACATGGTTCTACATGTCATTCATGAGGACTGATTG	TACGGTAGCAGAGACTTGGTCTAAAGCTGAAAATTTAGTTGGAAGG		
NF1	17	33	ACACTGACGACATGGTTCTACATTGGGAAGGTTAGAAACACTACCT	TACGGTAGCAGAGACTTGGTCTTGGATTTATGTGAAACCGAAA		
NF1	17	34	ACACTGACGACATGGTTCTACACAAGCCCTCCATATTTGTAATCT	TACGGTAGCAGAGACTTGGTCTAAAAGCACTATTCATGACCAATAA		
NF1	17	35	ACACTGACGACATGGTTCTACATGGTCCTGAGGTCTTTTTGG	TACGGTAGCAGAGACTTGGTCTTGGTGGCAAACTCTCCTTCT		
NF1	17	36	ACACTGACGACATGGTTCTACACTCAGTAGACAACATAAAGCCTCA	TACGGTAGCAGAGACTTGGTCTGCAGCTACTAGATACCGACCATC		
NF1	17	37.1	ACACTGACGACATGGTTCTACAAAAATAAAATTGATTAGTGGCATCTG	TACGGTAGCAGAGACTTGGTCTAGCCGCTCATGATACTTGGT	ACACTGACGACATGGTTCTACACATACCGGGCCTAGCAATC	TACGGTAGCAGAGACTTGGTCTGTGTACTCCCTGACCCAGGA
NF1	17	37.2	ACACTGACGACATGGTTCTACATTCCTGGCTTTGCTTACGAC	TACGGTAGCAGAGACTTGGTCTCACCTAGGGAGGCCAGGATA		
NF1	17	38.1	ACACTGACGACATGGTTCTACATCCACTTCACCCCGTCAC	TACGGTAGCAGAGACTTGGTCTTGCGATATTGAGCAGTGTCC		
NF1	17	38.2	ACACTGACGACATGGTTCTACATCACCTTAACCATTGCAAACC	TACGGTAGCAGAGACTTGGTCTCCAACACTGCATACCTTCCA		
NF1	17	39	ACACTGACGACATGGTTCTACATGTAACAGAATCACAAATTGTATGTTA	TACGGTAGCAGAGACTTGGTCTAAAGGGTTTTCTTTGAATTCTCTTA		
NF1	17	40	ACACTGACGACATGGTTCTACATTGATGTGATTTTCATTGACCA	TACGGTAGCAGAGACTTGGTCTGTGTCTAGCGCAGTGCTTTG		
NF1	17	41	ACACTGACGACATGGTTCTACATTGATTAGGCTGTTCCAATGAA	TACGGTAGCAGAGACTTGGTCTGGGACTCAAAGTTTTAGCACAA		
NF1	17	42	ACACTGACGACATGGTTCTACAAAAAAAAAAAATCCTGCTTCTTTAC	TACGGTAGCAGAGACTTGGTCTTGGTAAACACAAATTCCTTTCC		
NF1	17	43	ACACTGACGACATGGTTCTACAATTTTCTGTCTTTACTTGTTCCTTTA	TACGGTAGCAGAGACTTGGTCTCAAAATAGCACAATAAACCAATATTTC		
NF1	17	44	ACACTGACGACATGGTTCTACATGCATGGACTGTGTTATTGG	TACGGTAGCAGAGACTTGGTCTTGCAGGGATGGATTATATTGG		
NF1	17	45	ACACTGACGACATGGTTCTACAATGCATATTGTTGAAAATACAGCTA	TACGGTAGCAGAGACTTGGTCTATATTTCATTGACCTCAAATTTAAACG		
NF1	17	46	ACACTGACGACATGGTTCTACATCCTGAATTCATTCCGAGATTC	TACGGTAGCAGAGACTTGGTCTACTTCAAACAACTAAACTTTAAGAGG		
NF1	17	47	ACACTGACGACATGGTTCTACACCCCAAAAGAGAAAACATGG	TACGGTAGCAGAGACTTGGTCTGCAACAAGAAAAGATGGAAGAG		
NF1	17	48	ACACTGACGACATGGTTCTACATGTTCTGTGGTTTTCTGCAGTC	TACGGTAGCAGAGACTTGGTCTTCACTTATTCAAATTACTTCTGGTTTC		
NF1	17	49	ACACTGACGACATGGTTCTACACCTCAGCAGATGCTTGTTCA	TACGGTAGCAGAGACTTGGTCTCCACCACTAAAGGACTAGACTGTG		
NF1	17	50	ACACTGACGACATGGTTCTACATTCATCCTGTTTTAAGTCACACTTG	TACGGTAGCAGAGACTTGGTCTTCACTTACTCTTCCTAGGCCATC		
NF1	17	51	ACACTGACGACATGGTTCTACAAGGAAATAGGACAGCCACTTG	TACGGTAGCAGAGACTTGGTCTCATGGAAAATTTTGATAATCCTGA		
NF1	17	52	ACACTGACGACATGGTTCTACAGATGGAAAATAAAGGAAAGAAACTG	TACGGTAGCAGAGACTTGGTCTGACTTTCATGTACTCTCCCACCT		
NF1	17	53	ACACTGACGACATGGTTCTACAGGTGTTTGATCACGTTAATTCC	TACGGTAGCAGAGACTTGGTCTGTAGCCTGCCAAGATGCAA		
NF1	17	54	ACACTGACGACATGGTTCTACAGTCTTCTACTTCTCACCCAAACAGA	TACGGTAGCAGAGACTTGGTCTAACCCTCATTAAACCTACTGTCTCA		
NF1	17	55	ACACTGACGACATGGTTCTACAATCTCCCTTTAATTTTGGCACA	TACGGTAGCAGAGACTTGGTCTAGTCAGTGCATTCTACAACAGC		
NF1	17	56	ACACTGACGACATGGTTCTACAACTGTATGTCCAATGTAACTGGTTG	TACGGTAGCAGAGACTTGGTCTGTGTGTTCTTAAAGCAGGCATA		
NF1	17	57	ACACTGACGACATGGTTCTACATTAATATTTTTGGCTTCAGATGG	TACGGTAGCAGAGACTTGGTCTGTTGGTGTCTTATATTGTTGCTC		
NF1	17	58	ACACTGACGACATGGTTCTACAAGCGACACATGACTGCAATG	TACGGTAGCAGAGACTTGGTCTCAAACCGGATGGGTTCATTA		

B) RT-PCR Primers to amplify *FLT3* ITD

Gene Name	Chromosome	Exon #	
FLT3	13	13	TCCCTTTCATCCAAGACAACA
FLT3	13	16	TGAGTGCCTCTCTTTCAGAGC

References

1 Grossmann V, Kohlmann A, Zenger M, Schindela S, Eder C, Weissmann S, et al. A deep-sequencing study of chronic myeloid leukemia patients in blast crisis (BC-CML) detects mutations in 76.9% of cases. Leukemia : official journal of the Leukemia Society of America, Leukemia Research Fund, UK. 2011 Mar;25(3):557-60.

2 Haferlach C, Grossmann V, Kohlmann A, Schindela S, Kern W, Schnittger S, et al. Deletion of the tumor-suppressor gene NF1 occurs in 5% of myeloid malignancies and is accompanied by a mutation in the remaining allele in half of the cases. Leukemia : official journal of the Leukemia Society of America, Leukemia Research Fund, UK. 2012 Apr;26(4):834-9.

3 Grossmann V, Bacher U, Artusi V, Kohlmann A, Nadarajah N, Kern W, et al. Molecular analysis of RAS-RAF tyrosine-kinase signaling pathway alterations in patients with plasma cell myeloma. Blood cancer journal. 2012;2:e85.

Patient No.	Chromosome	Hg19 Genome Position (bp)	Gene	Exon	Mutation details: Reference allele	Alternative allele	DBSNP site	Diagnosis VAF (%)	Remission VAF (%)	Amino acid position	Reference amino acid	Mutated amino acid	Type of mutation	Polyphen2 Protein-damaging prediction	MutationTaster Protein-damaging prediction	SIFT Protein-damaging prediction	Sanger sequence validation	Targeted sequencing validation
1	chr1	17085006	MSTP9	11/15	C	T	DBSNP	8	3	490	G	E	Missense	Probably damaging	-			
1	chr1	215345428	KCNK2	5/7	C	T	NOVEL	60	0	227	A	V	Missense	Possibly damaging	Disease-causing mutation		Yes	
1	chr1	237586495	RYR2	12/105	G	A	NOVEL	41	0	318	D	N	Missense	Benign	Disease-causing mutation		Yes	
1	chr2	33768636	RASGRP3	12/17	G	A	NOVEL	39	0	445	E	K	Missense	Probably damaging	Disease-causing mutation		Yes	
1	chr2	77746691	LRRTM4	3/4	C	T	NOVEL	46	0	102	E	K	Missense	Benign	Disease-causing mutation		Yes	
1	chr3	33558652	CLASP2	34/38	G	A	NOVEL	51	0	1267	H	Y	Missense	Possibly damaging	Disease-causing mutation		Yes	
1	chr3	44929267	TGM4	3/14	C	T	NOVEL	50	0	94	Q	*	Frameshift	Probably damaging	Disease-causing mutation		Yes	
1	chr6	5613493	FARS2	6/7	G	A	NOVEL	11	0	386	R	Q	Missense	Probably damaging	Disease-causing mutation			
1	chr9	125054104	MRRF	5/7	G	A	NOVEL	50	0	179	R	Q	Missense	Possibly damaging	Disease-causing mutation			
1	chr12	6697096	CHD4	24/40	C	G	COSMIC	44	0	1162	R	P	Missense	Probably damaging	Disease-causing mutation		Yes	
1	chr12	22059189	ABCC9	10/38	C	T	NOVEL	48	0	497	E	K	Missense	Probably damaging	Disease-causing mutation		Yes	
1	chr12	26808679	ITPR2	20/57	G	A	NOVEL	41	0	851	P	S	Missense	Probably damaging	Disease-causing mutation			
1	chr12	55641421	OR6C74	1/1	C	T	NOVEL	43	0	117	S	F	Missense	Probably damaging	Disease-causing mutation		Yes	
1	chr12	62778031	USP15	10/21	G	A	NOVEL	10	0	445	R	H	Missense	Probably damaging	Disease-causing mutation			
1	chr12	79837985	SYT1	9/10	A	C	NOVEL	43	0	354	Q	P	Missense	Possibly damaging	Disease-causing mutation		Yes	
1	chr12	85257259-85257260	SLC6A15	11/12	CC	TT	NOVEL	47	0	593	G	R	Missense	Possibly damaging	Disease-causing mutation		Yes	
1	chr15	48784689	FBN1	24/66	C	T	NOVEL	47	0	941	M	I	Missense	Benign	Disease-causing mutation		Yes	
1	chr16	784854	NARFL	5/11	C	T	NOVEL	59	0	153	D	N	Missense	Probably damaging	Disease-causing mutation		Yes	
1	chr16	20998752	DNAH3	47/62	G	A	NOVEL	10	0	2301	R	W	Missense	Probably damaging	Disease-causing mutation			
1	chr16	24580488	RBBP6	17/18	G	A	COSMIC	9	0	826	R	H	Missense	Probably damaging	Disease-causing mutation			
1	chr17	29653001	NF1	37/58	C	T	NOVEL	35	0	1667	P	S	Missense	Probably damaging	Disease-causing mutation		Yes	
1	chr17	45234397	CDC27	7/19	G	A	DBSNP	13	0	242	P	S	Missense	Benign	Disease-causing mutation			
1	chr18	19031010	GREB1L	13/33	G	A	NOVEL	39	0	583	E	K	Missense	Probably damaging	Disease-causing mutation		Yes	
1	chr20	32996513	ITCH	4/25	G	A	NOVEL	67	0	43	V	I	Missense	Probably damaging	Disease-causing mutation		Yes	
1	chr20	55803435	BMP7	2/7	C	T	COSMIC	47	0	154	R	Q	Missense	Benign	Disease-causing mutation		Yes	
1	chrX	102977125	GLRA4	6/9	G	A	DBSNP	58	0	225	R	W	Missense	Probably damaging	Benign		Yes	
1	chrX	130409464	IGSF1	16/20	G	T	NOVEL	28	0	1058	L	I	Missense	Probably damaging	Disease-causing mutation			
3	chr1	162824712	C1orf110	4/4	G	C	DBSNP	4	0	251	S	*	Missense	Probably damaging	Disease-causing mutation			
3	chr1	240370120	FMN2	5/18	G	C	NOVEL	33	0	670	E	Q	Missense	Probably damaging	Disease-causing mutation			Yes
3	chr2	15607931	NBAS	-	G	A	NOVEL	22	0				Splice site		Disease-causing mutation			
3	chr2	37121091	STRN	7/18	C	G	NOVEL	25	0	294	S	*	Truncation		Disease-causing mutation			Yes
3	chr2	99013327	CNGA3	8/8	C	T	DBSNP, COSMIC	41	0	565	T	M	Missense	Probably damaging	Disease-causing mutation	Deleterious		
3	chr2	167136968	SCN9A	14/27	C	T	NOVEL	8	0	737	L	F	Missense	Probably damaging	Disease-causing mutation	Deleterious		
3	chr2	171811243	GORASP2	6/10	G	A	NOVEL	31	0	217	G	E	Missense	Benign	Disease-causing mutation	Deleterious		
3	chr3	46063244	XCR1	2/2	A	C	NOVEL	49	0	66	T	P	Missense	Probably damaging	Disease-causing mutation	Deleterious		Yes
3	chr3	88205168	C3orf38	-	C	G	NOVEL	26	0				Splice site		Disease-causing mutation			
3	chr4	89579597	HERC3	10/26	C	G	NOVEL	22	0	367	I	M	Missense	Possibly damaging	Disease-causing mutation	Deleterious		Yes
3	chr4	187535486	FAT1	12/27	G	A	NOVEL	15	0	3030	D	N	Missense	Benign	Disease-causing mutation			
3	chr4	187630726	FAT1	2/27	G	A	NOVEL	15	0	86	E	K	Missense	Probably damaging	Disease-causing mutation			Yes
3	chr5	111576501	EPB41L4A	10/23	G	A	DBSNP, COSMIC	45	0	268	E	K	Missense	Benign	Disease-causing mutation	Deleterious		
3	chr5	140746136	PCDHGA5	1/4	C	T	COSMIC	26	0	747	R	W	Missense	Possibly damaging	Disease-causing mutation	Deleterious		
3	chr7	40127785	CDK13	12/14	G	C	NOVEL	15	0	1030	Q	H	Missense	Probably damaging	Disease-causing mutation	Deleterious		
3	chr10	48371397	ZNF488	2/2	G	A	DBSNP	36	0	289	D	N	Missense	Probably damaging	Disease-causing mutation	Deleterious		
3	chr10	105377003	SH3PXD2A	10/14	G	A	NOVEL	37	0	263	V	I	Missense	Possibly damaging	Disease-causing mutation	Deleterious		Yes
3	chr11	63971536	STIP1	14/14	A	G	NOVEL	33	0	524	N	D	Splice site	Probably damaging	Disease-causing mutation	Deleterious		Yes
3	chr11	76796027	CAPN5	2/13	T	C	COSMIC	15	0	32	F	S	Missense	Possibly damaging	Disease-causing mutation	Deleterious		
3	chr12	20885882	SLCO1C1	10/15	C	T	NOVEL	40	0	409	S	F	Missense	Possibly damaging	Disease-causing mutation	Tolerated		Yes
3	chr12	26221734	RASSF8	5/5	G	A	NOVEL	25	0	415	E	K	Missense	Probably damaging	Disease-causing mutation	Deleterious		
3	chr13	32813860	FRY	46/61	G	A	NOVEL	17	0	2177	E	K	Missense	Probably damaging	Disease-causing mutation	Deleterious		Yes
3	chr13	33333821	PDS5B	29/35	C	T	NOVEL	20	0	1122	P	L	Missense	Possibly damaging	Disease-causing mutation	Tolerated		Yes
3	chr14	20404065	OR4K1	1/1	G	C	NOVEL	27	0	80	K	N	Missense	Probably damaging	Benign	Deleterious		Yes
3	chr14	54418601	BMP4	4/4	G	T	NOVEL	26	0	114	A	S	Missense	Probably damaging	Disease-causing mutation	Deleterious		
3	chr17	36830270	C17orf96	1/1	C	T	NOVEL	21	0	160	P	L	Missense	Probably damaging	Benign	Deleterious		Yes
3	chr19	34934777	UBA2	7/17	C	T	NOVEL	39	0	204	Q	*	Truncation		Disease-causing mutation			Yes
3	chr19	49982165	FLT3LG	1/68	G	GC	COSMIC	16	0	114-115	Q	X	Frameshift		Disease-causing mutation			
3	chr19	58151289	ZNF211	4/15	C	T	NOVEL	22	0	89	S	L	Missense	Possibly damaging	Benign	Deleterious		
3	chrX	17768017	SCML1	6/8	C	T	NOVEL	43	0	103	R	*	Truncation		Disease-causing mutation			Yes
3	chrX	107898629	COL4A5	37/53	G	T	NOVEL	50	0	1105	L	F	Missense	Benign	Disease-causing mutation			Yes
3	chrX	117707822	DOCK11	12/53	G	C	NOVEL	19	0	410	K	N	Missense	Possibly damaging	Disease-causing mutation	Deleterious		Yes
5	chr1	1275023	DVL1	10/15	C	G	NOVEL	28	0	331	I	M	Missense	Benign	Disease-causing mutation	Deleterious		Yes
5	chr1	153782716	GATAD2B	11/11	A	ACCCC	NOVEL	52	0	573	R	RGX	Frameshift		Disease-causing mutation			
5	chr1	171759675	METTL13	5/8	G	C	NOVEL	47	0	465	D	H	Missense	Probably damaging	Disease-causing mutation	Deleterious		Yes
5	chr1	173493929	SLC9C2	20/28	G	C	NOVEL	11	0	835	E	Q	Missense	Possibly damaging	Benign	Deleterious		
5	chr1	186294919	TPR	42/51	G	A	NOVEL	31	0	2030	R	K	Missense	Benign	Disease-causing mutation			Yes
5	chr1	208227783	PLXNA2	14/32	C	G	NOVEL	42	0	947	Q	E	Missense	Possibly damaging	Disease-causing mutation	Tolerated		Yes
5	chr2	170033060	LRP2	54/79	C	T	DBSNP	10	0	3478	H	Y	Missense	Probably damaging	Disease-causing mutation			
5	chr2	179474056	TTN	273/363	C	A	NOVEL	9	0	17327	S	R	Missense	Benign	Disease-causing mutation			
5	chr3	47161887-47161891	SETD2	3/21	ACTCT	A	NOVEL	52	0	1412-1413	ES	X	Frameshift		Disease-causing mutation			
5	chr3	52089905	DUSP7	1/3	A	G	NOVEL	43	0	160	K	E	Missense	Benign	Disease-causing mutation	Deleterious		Yes
5	chr3	99895209	CMSS1	9/10	G	A	NOVEL	35	0	236	D	N	Missense	Probably damaging	Disease-causing mutation	Deleterious		
5	chr3	110863745	PVRL3	-	G	A	NOVEL	41	0				Splice site		Disease-causing mutation			
5	chr3	113375194	KIAA2018	7/7	C	T	NOVEL	52	0	1779	Q	*	Splice site		Disease-causing mutation			
5	chr4	17841343	NCAPG	17/21	G	C	NOVEL	14	0	837	E	D	Missense	Probably damaging	Disease-causing mutation	Deleterious		
5	chr4	57876666	POLR2B	12/26	C	T	NOVEL	44	0	515	P	L	Missense	Probably damaging	Disease-causing mutation	Deleterious		Yes
5	chr4	76733470	USO1	24/25	C	T	NOVEL	8	0	923	A	V	Missense	Probably damaging	Disease-causing mutation			
5	chr4	123095753	KIAA1109	5/86	C	G	NOVEL	18	0	80	S	C	Missense	Probably damaging	Disease-causing mutation			Yes
5	chr4	177142616	ASB5	4/7	G	A	NOVEL	6	0	174	E	K	Truncation	Probably damaging	Disease-causing mutation	Deleterious		
5	chr5	173383124	CPEB4	10/10	C	A	NOVEL	30	0	725	S	*	Splice site		Disease-causing mutation			Yes
5	chr6	144081539	PHACTR2	5/13	G	A	NOVEL	38	0	152			Splice site		Disease-causing mutation			
5	chr7	11500386	THSD7A	11/28	G	C	COSMIC	6	0	836	K	N	Missense	Probably damaging	Disease-causing mutation	Deleterious		
5	chr7	138204006	TRIM24	4/19	G	A	NOVEL	55	0	235	C	Y	Missense	Probably damaging	Disease-causing mutation	Deleterious		Yes
5	chr7	138235907	TRIM24	8/19	C	T	NOVEL	33	0	415	Q	*	Splice site		Disease-causing mutation			Yes
5	chr7	138749702	ZC3HAV1	8/13	A	T	COSMIC	67	0	639	E	V	Missense	Benign	Disease-causing mutation	Deleterious		Yes
5	chr8	76465357	HNF4G	5/10	G	C	COSMIC	50	0	180	M	I	Missense	Possibly damaging	Disease-causing mutation	Tolerated		Yes
5	chr9	79635850	FOXB2	1/1	C	T	COSMIC	11	0	427	S	L	Missense	Probably damaging	Benign	Deleterious		
5	chr9	125377069	OR1O1	1/1	C	G	NOVEL	11	0	18	S	C	Missense	Probably damaging	Benign	Deleterious		
5	chr9	130885372	PTGES2	5/7	T	G	NOVEL	18	0	243	V	G	Missense	Probably damaging	Disease-causing mutation	Deleterious		
5	chr10	64952712	JMJD1C	16/26	G	C	NOVEL	45	0	2021	R	T	Missense	Probably damaging	Disease-causing mutation	Deleterious		Yes
5	chr10	90697935	ACTA2	8/9	C	G	NOVEL	13	0	291	I	M	Missense	Probably damaging	Disease-causing mutation	Deleterious		
5	chr11	17741913	MYOD1	1/3	G	A	NOVEL	7	0	195	G	D	Missense	Probably damaging	Disease-causing mutation	Deleterious		
5	chr11	57428405	CLP1	3/3	C	G	NOVEL	52	0	259	Q	E	Missense	Possibly damaging	Disease-causing mutation	Deleterious		Yes
5	chr11	64822077	NAALADL1	5/18	G	A	COSMIC	33	0	246	R	Q	Missense	Probably damaging	Disease-causing mutation	Deleterious		Yes
5	chr11	72632974	FCHSD2	9/20	G	T	NOVEL	28	0	243	D	Y	Missense	Possibly damaging	Disease-causing mutation	Deleterious		Yes
5	chr11	76796027	CAPN5	2/13	T	C	COSMIC	15	0	32	F	S	Missense	Possibly damaging	Disease-causing mutation	Deleterious		
5	chr11	94533290	AMOTL1	3/13	G	C	NOVEL	30	0	312	E	Q	Missense	Probably damaging	Disease-causing mutation	Tolerated		Yes
5	chr11	119103299	CBL	2/16	G	C	NOVEL	21	0	113	E	Q	Missense	Possibly damaging	Disease-causing mutation	Deleterious		Yes
5	chr12	29689147	TMTC1	11/18	G	C	NOVEL	8	0	594	E	Q	Missense	Probably damaging	Disease-causing mutation	Tolerated		
5	chr12	54912519	NCKAP1L	-	A	G	NOVEL	5	0				Splice site		Disease-causing mutation			
5	chr12	75785038	GLIPR1L2	1/6	G	A	COSMIC	20	0	48	E	K	Missense	Probably damaging	Disease-causing mutation	Tolerated		
5	chr12	80014945	PAWR	3/7	C	T	DBSNP	10	0	187	R	W	Missense	Probably damaging	Disease-causing mutation	Deleterious		
5	chr12	112888198	PTPN11	3/16	G	A	DBSNP	67	0	72	A	T	Missense	Probably damaging	Disease-causing mutation	Deleterious		Yes
5	chr12	124366181	DNAH10	50/78	G	A	NOVEL	32	0	2764	E	K	Missense	Possibly damaging	Disease-causing mutation		Yes	
5	chr15	52017212	LYSMD2	2/3	G	C	NOVEL	10	0	127	G	A	Missense	Probably damaging	Benign	Deleterious		
5	chr17	15641610	TBC1D26	7/15	A	G	DBSNP	20	0	99	Y	C	Missense	Possibly damaging	Benign	Deleterious		
5	chr17	78064000-78064064	CCDC40	18/18	AGGCACG	A	NOVEL	67	0	966-987	GTCTKNTG	X	Frameshift		Disease-causing mutation			
5	chr19	38103007	ZNF540	5/5	T	C	NOVEL	50	0	276	C	R	Missense	Probably damaging	Disease-causing mutation	Deleterious		Yes
5	chrX	70344126	MED12	13/45	G	A	NOVEL	17	0	621	R	Q	Missense	Probably damaging	Disease-causing mutation			Yes
5	chrX	109694000	RGAG1	3/4	C	T	NOVEL	7	0	52	S	L	Missense	Possibly damaging	Benign	Deleterious		
5	chrX	117119202	KLHL13	-	C	A	NOVEL	38	0				Splice site		Disease-causing mutation			
5	chrX	154743751	TMLHE	4/8	G	C	NOVEL	48	0	178	K	N	Missense	Probably damaging	Disease-causing mutation	Deleterious		Yes
7	chr1	981434	AGRN	16/36	C	T	DBSNP	53	0	924	P	L	Missense	Possibly damaging	Disease-causing mutation	Deleterious		Yes
7	chr1	7887421	PER3	18/19	C	T	NOVEL	46	0	803	P	L	Missense	Possibly damaging	Benign	Deleterious		Yes
7	chr1	36563558	COL8A2	2/2	G	A	DBSNP	36	0	575	P	L	Missense	Probably damaging	Disease-causing mutation	Deleterious		
7	chr1	52489198	TXNDC12	6/7	G	A	NOVEL	45	0	135	P	L	Missense	Possibly damaging	Disease-causing mutation	Deleterious		Yes
7	chr1	151633333	SNX27	4/12	G	T	NOVEL	12	0	173	D	Y	Missense	Probably damaging	Disease-causing mutation	Deleterious		
7	chr1	237755118	RYR2	32/105	C	T	NOVEL	36	0	1412	R	W	Missense	Possibly damaging	Benign			Yes
7	chr2	25678304	DTNB	10/18	G	A	NOVEL	48	0	176	S	F	Missense	Benign	Disease-causing mutation			Yes
7	chr5	13766107	DNAH5	2/21	C	T	COSMIC	47	0	3360	G	E	Missense	Probably damaging	Disease-causing mutation			Yes
7	chr5	98209411	CHD1	1/10	G	A	COSMIC	43	0	1153	R	*	Truncation		Disease-causing mutation			Yes
7	chr6	25653624	SCGN	2/11	G	A	NOVEL	38	0	33	E	K	Missense	Benign	Disease-causing mutation	Deleterious		Yes
7	chr7	938780	ADAP1	11/12	G	A	NOVEL	62	0	329	T	M	Missense	Probably damaging	Disease-causing mutation	Deleterious		Yes
7	chr7	2649749	IOCE	22/22	G	A	NOVEL	31	0	630	D	N	Missense	Benign	Disease-causing mutation	Deleterious		Yes
7	chr8	144940290	EPPK1	1/1	C	G	DBSNP, COSMIC	18	0	2378	D	H	Missense	Probably damaging	Disease-causing mutation			
7	chr10	108468976	SORCS1	-	C	T	NOVEL	42	0				Splice site		Disease-causing mutation			
7	chr11	15134025	INSC	1/13	C	A	NOVEL	10	0	4	P	T	Missense	Probably damaging	Benign	Deleterious		
7	chr11	66189919	NPAS4	-	C	T	NOVEL	41	0				Splice site		Disease-causing mutation			
7	chr11	92881990	SLC36A4	10/10	G	A	NOVEL	45	0	275	P	S	Missense	Probably damaging	Disease-causing mutation	Deleterious		Yes
7	chr11	102033222	YAP1	3/9	G	A	NOVEL	32	0	203	R	K	Missense	Benign	Disease-causing mutation	Deleterious		Yes
7	chr12	14018971	GRIN2B	2/13	C	T	NOVEL	43	0	58	D	N	Missense	Probably damaging	Disease-causing mutation	Tolerated		Yes
7	chr12	56722350	PAN2	3/26	G	A	NOVEL	70	0	120	R	W	Missense	Probably damaging	Disease-causing mutation	Deleterious		Yes
7	chr12	120784071	MSI1	3/26	C	T	NOVEL	29	0	218	G	E	Missense	Probably damaging	Disease-causing mutation	Deleterious		Yes
7	chr13	23909764	SACS	8/8	C	T	NOVEL	36	0	2001	E	K	Missense	Probably damaging	Disease-causing mutation	Tolerated		Yes
7	chr13	35770072	NBEA	30/57	G	A	NOVEL	23	0	1667	E	K	Missense	Benign	Disease-causing mutation			Yes
7	chr13	77642818	MYCBP2	12/25	G	A	NOVEL	36	0	4018	S	F	Missense	Probably damaging	Disease-causing mutation			Yes
7	chr14	78285427	ADCK1	2/9	G	A	NOVEL	49	0	36	A	T	Missense	Possibly damaging	Disease-causing mutation	Tolerated		Yes
7	chr14	105054119	C14orf180	3/5	A	G	NOVEL	58	0	49	K	E	Missense	Possibly damaging	Benign	Deleterious		
7	chr15	72185407	MYO9A	3/19	C	T	NOVEL	21	0	1752	D	N	Missense	Probably damaging	Disease-causing mutation			Yes
7	chr16	52484232	TOX3	4/7	G	A	NOVEL	42	0	212	P	L	Missense	Probably damaging	Disease-causing mutation	Deleterious		Yes
7	chr19	13409572	CACNA1A	10/38	G	A	NOVEL	51	0	960	R	C	Missense	Probably damaging	Disease-causing mutation			Yes
7	chr19	40730629	CNTD2	2/5	G	A	NOVEL	28	0	13	H		Splice site		Disease-causing mutation			
7	chr19	51512524	KLK9	2/7	C	T	NOVEL	60	0	39	G	S	Missense	Probably damaging	Disease-causing mutation	Deleterious		Yes
7	chr20	39788792	PLCG1	-	C	T	COSMIC	49	0				Splice site	Possibly damaging	Disease-causing mutation			Yes
7	chr22	37906238	CARD10	5/25	A	C	NOVEL	50	0	297	L	W	Missense	Possibly damaging	Disease-causing mutation	Deleterious		
7	chrX	2942083	ARSH	6/9	A	C	NOVEL	50	0	308	D	A	Missense	Possibly damaging	Disease-causing mutation	Deleterious		Yes
7	chrX	117773465	DOCK11	38/53	C	T	NOVEL	33	0	1357	R	*	Truncation		Disease-causing mutation			Yes
7	chrX	151366218	GABRA3	8/10	C	T	COSMIC	48	0	273	R	Q	Missense	Probably damaging	Disease-causing mutation	Deleterious		Yes
9	chr1	115258748	NRAS	2/7	G	A	COSMIC	6	0	12	G	S	Missense	Benign	Disease-causing mutation	Deleterious	Yes	
9	chr1	207013267	IL19	4/6	G	T	NOVEL	9	0	133	E	*	Truncation		Disease-causing mutation			
9	chr1	215960153	USH2A	52/72	T	G	COSMIC	44	0	3416	C	G	Missense	Probably damaging	Disease-causing mutation			
9	chr2	63223884	EHBP1	21/25	G	A	NOVEL	15	0	1100	R	H	Missense	Probably damaging	Disease-causing mutation	Deleterious		Yes
9	chr3	175345098	NAALADL2	11/14	A	G	NOVEL	8	0	607	E	G	Missense	Probably damaging	Disease-causing mutation	Deleterious		
9	chr4	88535832	DSPP	4/4	A	ATAGCAG	NOVEL	42	0	673	D	DSSDSSS	Inframe insertion		Disease-causing mutation			
9	chr5	88027592	MEF2C	9/12	T	A	NOVEL	48	0	273	L	H	Missense	Probably damaging	Disease-causing mutation	Deleterious		Yes
9	chr6	47647949	GPR111	5/6	C	A	NOVEL	46	0	205	P	H	Missense	Probably damaging	Benign	Deleterious		Yes
9	chr7	45009048	MYO1G	7/17	G	T	DBSNP	25	0	513	R	L	Missense	Probably damaging	Disease-causing mutation	Deleterious		
9	chr8	145541368	DGAT1	11/17	T	G	NOVEL	20	0	301	V	G	Missense	Probably damaging	Disease-causing mutation	Deleterious		
9	chr9	4118418	GLIS3	4/11	G	A	NOVEL	41	0	354	V	M	Missense	Probably damaging	Disease-causing mutation	Deleterious		Yes
9	chr9	72895687	SMC5	6/25	G	T	NOVEL	7	0	231	E	*	Truncation		Disease-causing mutation			
9	chr10	117075090	ATRNL1	18/29	T	G	COSMIC	18	0	961	C	G	Missense	Probably damaging	Disease-causing mutation	Deleterious		
9	chr12	23689454	SOX5	14/15	C	T	COSMIC	49	0	641	R	C	Missense	Probably damaging	Disease-causing mutation	Deleterious		Yes
9	chr12	43846379	ADAMTS20	13/39	G	A	NOVEL	42	0	627	G	D	Missense	Probably damaging	Disease-causing mutation	Deleterious		Yes
9	chr13	72049909	DACH1	9/11	C	T	NOVEL	35	0	650	T	M	Missense	Possibly damaging	Disease-causing mutation			Yes
9	chr14	38723768	CLEC14A	1/1	C	T	NOVEL	46	0	487	S	F	Missense	Probably damaging	Benign	Deleterious		Yes
9	chr15	42968113	STARD9	20/33	A	G	COSMIC	13	0	605	D	G	Missense	Possibly damaging	Benign			
9	chr15	100801792	ADAMTS17	6/22	G	A	NOVEL	12	0	308	C	Y	Missense	Probably damaging	Disease-causing mutation	Deleterious		
9	chr19	49982165	FLT3LG	1/68	G	GC	COSMIC	28	0	114-115	Q	X	Frameshift		Disease-causing mutation			
21	chr1	104076466	RNPC3	3/15	GA	G	COSMIC	15	0	116	E	X	Frameshift		Disease-causing mutation			
21	chr8	144874523	SCRIB	32/37	G	A	NOVEL	8	0	1461	A	T	Missense	Probably damaging	Disease-causing mutation			
21	chr11	1017183	MUC6	31/33	C	A	COSMIC	16	0	1873	P	Q	Missense	Probably damaging	Benign			
21	chr12	10588530	KLRC2	1/6	C	G	COSMIC	29	0	19	R	P	Missense	Probably damaging	Benign	Deleterious		
21	chr19	12739215	ZNF791	4/4	A	C	NOVEL	7	0	291	E	A	Missense	Benign	Disease-causing mutation	Deleterious		

43	chr1	153233991	LOR	2/2	A	ACTCTGG	COSMIC	32	0	189			Splice site		Disease-causing mutation			
43	chr2	230456417	DNER	2/13	C	A	COSMIC	41	0	155	P	L	Missense	Benign	Disease-causing mutation	Deleterious		Yes
43	chr4	126329626	FAT4	4/17	C	T	DBSNP	38	0	1866	T	M	Missense	Probably damaging	Disease-causing mutation			
43	chr5	141039044	ARAP3	-	C	A	NOVEL	39	0				Splice site		Disease-causing mutation			
43	chr5	178416050	GRM6	6/10	G	T	NOVEL	41	0	414	A	T	Missense	Probably damaging	Disease-causing mutation	Deleterious		Yes
43	chr11	108100021	ATM	4/63	T	A	NOVEL	71	0	101	V	D	Missense	Probably damaging	Disease-causing mutation			Yes
43	chr12	53680313	ESPL1	18/31	C	G	NOVEL	42	0	1265	R	G	Missense	Possibly damaging	Disease-causing mutation	Deleterious		Yes
43	chr13	28602340	FLT3	16/24	C	T	COSMIC	26	0	676	N	K	Missense	Probably damaging	Disease-causing mutation	Deleterious		Yes
43	chr14	93301915	GOLGA5	11/13	C	T	NOVEL	36	0	653	R	*	Truncation		Disease-causing mutation			Yes
43	chr14	100604072	EVL	-	C	T	DBSNP	34	0				Splice site		Disease-causing mutation			
43	chr17	7577093	TP53	8/11	G	C	COSMIC	78	0	282	R	P	Missense	Probably damaging	Disease-causing mutation	Deleterious		Yes
43	chr17	76082940	TNRC6C	13/21	C	T	NOVEL	41	0	1187	R	C	Missense	Probably damaging	Disease-causing mutation	Deleterious		Yes
43	chr19	42860512	MEGF8	25/41	G	A	DBSNP	44	0	1443	R	H	Missense	Probably damaging	Disease-causing mutation	Deleterious		Yes
43	chr22	43985982	EFCAB6	24/32	G	T	COSMIC	41	0	1002	E	K	Missense	Possibly damaging	Benign	Deleterious		Yes
43	chrX	47836179	ZNF182	7/7	G	A	NOVEL	9	0	436	G	V	Missense	Probably damaging	Disease-causing mutation	Deleterious		
44	chr1	63877638	ALG6	9/15	C	T	NOVEL	10	0	241	S	F	Missense	Possibly damaging	Disease-causing mutation	Deleterious		
44	chr1	115258748	NRAS	2/7	G	T	COSMIC	39	0	12	G	S	Missense	Benign	Disease-causing mutation	Deleterious	Yes	
44	chr2	84934670	DNAH6	54/77	C	T	NOVEL	13	0	2960	R	C	Missense	Probably damaging	Disease-causing mutation	Deleterious		
44	chr3	122545724	DIRC2	3/9	C	T	DBSNP	49	0	172	T	M	Missense	Benign	Disease-causing mutation	Deleterious		Yes
44	chr3	125850318	ALDH1L1	13/23	C	A	COSMIC	50	0	521	A	V	Missense	Possibly damaging	Disease-causing mutation	Deleterious		Yes
44	chr4	147560457-147560466	POU4F2	1/2	TGGCGGC	TGGCGGC	NOVEL	41	0	55-58			Splice site, indel		Disease-causing mutation			
44	chr5	140769385	PCDHGB4	1/4	G	A	NOVEL	39	0	645	R	H	Missense	Benign	Disease-causing mutation	Deleterious		
44	chr6	16327915-16327918	ATXN1	8/9	ATGC	ATGCTGC	NOVEL	100	0	208-209	OH	OOOH	Inframe indel		Disease-causing mutation			
44	chr9	19395661-19395669	HRCT1	1/1	CTCCTTC	CTCCTTC	NOVEL	100	0	100-101			Splice site, indel		Disease-causing mutation			
44	chr12	11214601	TAS2R46	1/1	T	G	NOVEL	8	0	98	L	P	Missense	Possibly damaging	Disease-causing mutation	Deleterious		
44	chr14	92537353	ATXN3	10/11	C	CG	NOVEL	45	0	315	G	AX	Frameshift		Disease-causing mutation			
44	chr15	89074487	DET1	3/6	C	T	NOVEL	8	0	161	D	E	Missense	Possibly damaging	Disease-causing mutation	Tolerated		
44	chr19	33490585	RHPN2	10/15	C	A	DBSNP, COSMIC	17	0	378	O	*	Truncation		Disease-causing mutation			

Supplementary Table 4: A list of mutations in eight iAMP21 patient samples. WES was performed on the matched diagnostic and remission material of eight iAMP21 patient samples (patients 1, 3, 5, 7, 9, 21, 43 and 44). Somatic mutations that were predicted to alter protein function by the majority of protein-damaging predition tools (PolyPhen2, SIFT and Mutation Taster) are listed. DBSNP variants with <1% minor allelic frequency (MAF) were included. Mutations in components of the RAS pathway are highlighted in grey.

Supplelmentary Table 5: RAS pathway mutations in iAMP21-ALL. 44 mutations were identified in 26 (25 diagnostic and 1 relapse) iAMP21-ALL patient samples (column A); relapse samples are depicted by 'b', as shown in Supplementary Table 1. Details of the mutation (columns B-D, F), genomic location of the variation (column E) and the predicted functional consequence of the mutation, as predicted by Mutation Taster and Polyphen2 (columns G-I), are shown. Mutations previously identified in cancer are highlighted (column J); yes* defines *FLT3*- ITD mutations that have been previously reported but the number of inserted amino acids is different.

Patient #	Gene	Mutation - amino acid change	Variant allele frequency (%)	Genome position	Nucleotide change	Type of mutation	Polyphen2 Protein-damaging	MutationTaster Protein-damaging	Reported in COSMIC
1	*NRAS*	Q22K	5%	chr1:115258718	C/A	Missense	Probably damaging	Disease-causing mutation	Yes
1	*NF1*	P1667S	39%	chr17:29653001	C/T	Missense	Probably damaging	Disease-causing mutation	No
3	*KRAS*	L19F	2%	chr12:25398262	G/C	Missense	Probably damaging	Disease-causing mutation	Yes
3	*NRAS*	E49K	3%	chr1:115256566	C/T	Missense	Benign	Disease-causing mutation	Yes
3	*NRAS*	R68T	3%	chr1:115256508	C/G	Missense	Probably damaging	Disease-causing mutation	Yes
3	*PTPN11*	E76Q	4%	chr12:112888210	G/C	Missense	Probably damaging	Disease-causing mutation	Yes
4	*NRAS*	G13D	29%	chr1:115258744	C/T	Missense	Benign	Disease-causing mutation	Yes
5	*PTPN11*	N58S	48%	chr12:112888157	A/G	Missense	Possibly damaging	Disease-causing mutation	Yes
5	*PTPN11*	A72T	44%	chr12:112888198	G/A	Missense	Probably damaging	Disease-causing mutation	Yes
6	*FLT3*	L610>WAREYEYDLKWEFPRENL	13%	chr13:28608251	51bp insertion	Indel	NA	NA	Yes
7	*FLT3*	P606>IWEYDLKWEF	4%	chr13:28608239	30bp insertion	Indel	NA	NA	Yes
8	*NRAS*	R68fs	6%	chr1:115256506	GAGA/TTCC	Indel	NA	Disease-causing mutation	No
8	*FLT3*	D835V	3%	chr13:28592641	T/A	Missense	Probably damaging	Disease-causing mutation	Yes
9	*NRAS*	G12S	8%	chr1:115258748	C/T	Missense	Possibly damaging	Disease-causing mutation	Yes
9	*NRAS*	G12C	2%	chr1:115258748	C/A	Missense	Possibly damaging	Disease-causing mutation	Yes
9	*NRAS*	G12D	2%	chr1:115258747	C/T	Missense	Benign	Disease-causing mutation	Yes
9	*NRAS*	G13D	5%	chr1:115258744	C/T	Missense	Benign	Disease-causing mutation	Yes
10	*KRAS*	G12D	37%	chr12:25398284	C/T	Missense	Possibly damaging	Disease-causing mutation	Yes
10	*KRAS*	G12D	5%	chr12:25398284	C/T	Missense	Possibly damaging	Disease-causing mutation	Yes
11	*FLT3*	F605>SRRYEYDLKWEF	9%	chr13:28608242	33bp insertion	Indel	NA	NA	Yes*
12	*FLT3*	F590fs	9%	chr13:28608287	10bp insertion	Frameshift	NA	Disease-causing mutation	Yes*
12b	*NRAS*	G12D	48%	chr1:115258747	C/T	Missense	Benign	Disease-causing mutation	Yes
12b	*PTPN11*	R351Q	48%	chr12:112915779	G/A	Missense	Benign	Disease-causing mutation	No
14	*NRAS*	Q61H	31%	chr1:115256528	T/A	Missense	Benign	Disease-causing mutation	Yes
17	*PTPN11*	E76K	35%	chr12:112888210	G/A	Missense	Probably damaging	Disease-causing mutation	Yes
18	*NRAS*	Y64_S65insGQEE	42%	chr1:115256521	12bp insertion	Indel	NA	Disease-causing mutation	No
19	*NRAS*	G12S	21%	chr1:115258748	C/T	Missense	Benign	Disease-causing mutation	Yes
19	*NRAS*	G12V	24%	chr1:115258747	C/A	Missense	Possibly damaging	Disease-causing mutation	Yes
20	*NRAS*	G13V	5%	chr1:115258744	C/A	Missense	Probably damaging	Disease-causing mutation	Yes
20	*NRAS*	Y64N	3%	chr1:115256521	A/T	Missense	Probably damaging	Disease-causing mutation	Yes
21	*KRAS*	G12V	11%	chr12:25398284	C/A	Missense	Probably damaging	Disease-causing mutation	Yes
21	*FLT3*	K602>LADEYFYVDFREYEYDL	13%	chr13:28608252	51bp insertion	Indel	NA	NA	Yes
22	*KRAS*	G12R	26%	chr12:25398285	C/G	Missense	Possibly damaging	Disease-causing mutation	Yes
22	*KRAS*	G12V	10%	chr12:25398284	C/A	Missense	Possibly damaging	Disease-causing mutation	Yes
24	*FLT3*	I836del	6%	chr13:28592637	3bp deletion	Indel	NA	Disease-causing mutation	Yes
26	*FLT3*	F612>LDFREYEYDLKWEFPRENLEF	16%	chr13:28608220	60bp insertion	Indel	NA	NA	Yes*
29	*NRAS*	G12S	48%	chr1:115258748	C/T	Missense	Benign	Disease-causing mutation	Yes
30	*NRAS*	G13D	12%	chr1:115258744	C/T	Missense	Benign	Disease-causing mutation	Yes
30	*NRAS*	G13V	6%	chr1:115258744	C/A	Missense	Probably damaging	Disease-causing mutation	Yes
31	*KRAS*	G13D	52%	chr12:25398281	C/T	Missense	Possibly damaging	Disease-causing mutation	Yes
31	*KRAS*	F78L	2%	chr12:25380226	A/G	Missense	Possibly damaging	Disease-causing mutation	No
37	*FLT3*	N609>RVLSGHVDFREYEYDLKWEFPREN	9%	chr13:28608230	69bp insertion	Indel	NA	NA	Yes*
39	*NRAS*	Q61L	2%	chr1:115256529	T/A	Missense	Probably damaging	Disease-causing mutation	Yes
39	*BRAF*	V600E	44%	chr7:140453136	A/T	Missense	Probably damaging	Disease-causing mutation	Yes

Patient sample	Diagnostic lesion	Relapse lesion
6[BD]	*RB1* deletion (CN=1)<br>*IKZF1* deletion (CN=1)<br>*FLT3* ITD (L610>E611ins18) (VAF, 13%)	*IKZF1* deletion (CN=1)<br>*CRLF2* rearrangement
12[ABC]	*IKZF1* deletion (CN=1)<br>*CRLF2* gain (CN=3)<br>*FLT3* F590_D593fs (VAF, 9%)<br>Ch11:96439395-134944770 (CN=1)	*IKZF1* deletion (CN=1)<br>*EBF1* gain (CN=3)<br>*CRLF2* rearrangement<br>*NRAS* G12D (VAF, 48%)<br>*PTPN11* R351Q (VAF, 48%)<br>Ch11:96439395-134944770 (CN=1)
24[AD]	*FLT3* I836>M<br>Ch7:120150796-159119708 (CN=1)	Ch7:120150796-159119708 (CN=1)
33[BC]	*RB1* deletion (CN=1)	
34[BC]		*ETV6* deletion (CN=1)
35[BC]	*ETV6* deletion (CN=1)	*IKZF1* deletion (CN=1)
36[BD]	*RB1* deletion (CN=0)<br>*CRLF2* gain (CN=3)	*RB1* deletion (CN=0)

Supplementary Table 6: Summary of genomic abnormalities in 7 matched diagnostic and relapse iAMP21-ALL samples. Seven diagnostic and relapse matched pairs were interrogated by SNP6·0 array ([A]) (to identify copy number abnormalities (CNA) of RAS pathway gene loci), MLPA ([B]) (to identify CNAs of recurrently deleted ALL genes), and targeted sequencing approaches (Screen A ([C]) and Screen B ([D])) (Supplementary Figure 1). All cases were confirmed as iAMP21-ALL at diagnosis and relapse (Supplementary Table 1). The genomic aberrations that were similar at diagnosis and relapse are highlighted in red text. Mutations and CNA identified by MLPA were not detected in the diagnostic sample of patient 34 or the relapse material of patient 33. A single CNA or somatic mutation was not consistent or specific to diagnostic and/or relapse iAMP21-ALL. The genomic profile was frequently different between the 7 matched diagnostic and relapse samples. Abbreviations: VAF, variant allele frequency; CN, gene copy number.
